# The indirect association between psychological resilience and prosocial behavior through cognitive reappraisal in Chinese college students

**DOI:** 10.3389/fpsyg.2026.1789130

**Published:** 2026-09-07

**Authors:** Chen Jing, Amalia Bt Madihie, Ren Yuqing, Qin Chao

**Affiliations:** 1 Universiti Malaysia Sarawak, Kota Samarahan, Malaysia; 2 Guizhou Industrial Vocational and Technical University, Guiyang, China; 3 No. 22 Kindergarten of Baiyun District, Guiyang, China; 4 Weifang Institute of Technology, Weifang, China

**Keywords:** college students, emotion reappraisal, mediating role, prosocial behavior, psychological resilience, resilience

## Abstract

**Introduction:**

Psychological resilience is commonly defined as an individual’s capacity to maintain stability and engage in constructive adaptation in the face of adversity, and its role in promoting positive developmental outcomes among college students has received increasing attention. Individuals with higher levels of resilience tend to exhibit more balanced emotional responses under stress, which may in turn facilitate their involvement in prosocial actions such as helping, comforting, cooperating, sharing, and donating. Cognitive reappraisal-the cognitive reframing of potentially stressful situations-is believed to further reinforce this adaptive response by mitigating the adverse effects of stress. However, the structural nature of the interrelations among psychological resilience, cognitive reappraisal, and prosocial behavior remains insufficiently understood, particularly regarding whether cognitive reappraisal accounts for part of the resilience–prosocial behavior association in cross-sectional data. The present study, therefore, aimed to examine the statistical dependencies among these variables based on cross-sectional survey data, with a view to providing empirically grounded references for psychological health education among college students.

**Methods:**

A cross-sectional survey was administered to 411 college students recruited from eight universities across four cities in Guizhou Province, China. Participants completed three self-report instruments: the Psychological Resilience Questionnaire (CD-RISC), the Prosocial Behavior Tendency Questionnaire for College Students (PTM), and the Emotion Regulation Strategies Questionnaire (ERQ). All measures have been previously validated in Chinese samples. Data were analyzed using SPSS 22.0. Correlation and regression analyses were conducted to examine bivariate associations among the variables. To estimate the statistical indirect effect, the PROCESS macro for SPSS (Model 4; Hayes et al., 2018) was used with 5,000 bootstrap resamples to generate bias-corrected 95% confidence intervals. Model fit for the proposed structure was evaluated using structural equation modeling in AMOS. In addition, three competing models the proposed model, a reverse-path model, and a common-cause model were compared to assess the extent to which the cross-sectional data could distinguish between alternative directional explanations.

**Results:**

Correlation analyses showed that the psychological resilience was positively correlated with prosocial behavior (*r* = 0.469, *p* < 0.001), and cognitive reappraisal was also positively correlated with prosocial behavior (*r* = 0.474, *p* < 0.001). Regression analysis indicated that psychological resilience accounted for 22.0% of the variance in prosocial behavior (*R*^2^ = 0.220, adjusted *R*^2^ = 0.218), with an unstandardized coefficient of 0.490 (*t* = 10.732, *p* < 0.001). When regressed on cognitive reappraisal, psychological resilience explained 34.4% of the variance (*R*^2^ = 0.344, adjusted *R*^2^ = 0.343), with a coefficient of 0.202 (*t* = 14.659, *p* < 0.001). The indirect effect of psychological resilience on prosocial behavior through cognitive reappraisal was estimated at 0.180 [BootSE = 0.041; 95% BootCI (0.100, 0.263)], accounting for approximately 38% of the total effect. When cognitive reappraisal was included in the model, the direct effect of resilience on prosocial behavior decreased from 0.471 to 0.292 (*t* = 5.596, *p* < 0.001), while the path from reappraisal to prosocial behavior remained significant (β = 0.214, *t* = 5.862, *p* < 0.001). The proposed model demonstrated good fit (CFI = 0.993, RMSEA = 0.044). However, comparison with competing models including a reverse-path model (Reappraisal → Resilience → Prosocial Behavior) and a common-cause model showed that all three models produced nearly identical fit indices, with indirect effect estimates of 0.180 for the proposed model and 0.183 for the reverse model. This equivalence is a mathematical consequence of the cross-sectional design and indicates that the data cannot distinguish between directional alternatives.

**Discussion:**

In the current college student sample, both psychological resilience and cognitive reappraisal were positively correlated with prosocial behavior, and cognitive reappraisal accounted for a portion of the statistical effect underlying the psychological resilience–prosocial behavior association within the same cross-sectional data. This finding suggests the presence of statistical conditional dependencies among resilience, emotion regulation strategies, and prosocial tendencies; however, the nature of these interrelations should be interpreted with caution, given the cross-sectional design of the present study.

## 1 Introduction

Garmezy et al. (2012) describes psychological resilience as “the ability of individuals to with stand adverse environmental pressures and make adjustments for better development” (p. 23). This process-oriented definition frames resilience as a dynamic developmental trajectory in which individuals achieve positive adaptation following adversity. It reflects ongoing interactions between individuals and their environments and highlights the role of personal characteristics in maintaining psychological balance. Across different conceptualizations, two common elements emerge: exposure to adversity and successful adaptation leading to positive developmental outcomes. In line with this perspective, the present study adopts a process-oriented view, conceptualizing psychological resilience as the capacity for effective adaptation to environmental challenges.

Prosocial behavior is generally understood as voluntary action intended to benefit others or society, including behaviors such as helping, sharing, and providing emotional support (Scigala et al., 2022). Empirical studies have shown that prosocial tendencies are shaped by a combination of situational factors and individual differences, including moral reasoning and emotional responsiveness. For instance, Lozada et al. (2021) found that higher levels of prosocial behavior were associated with better socio-emotional adjustment, as reflected in stronger interpersonal bonds and more cooperative group dynamics. Similarly, research by Khadka (2024) indicated that individuals who reported stronger identification with their social groups were more likely to engage in behaviors that supported collective goals, suggesting a link between social identity and prosocial action.

Prior research has begun to examine how internal psychological resources relate to prosocial behavior. Individuals with higher psychological resilience tend to report more adaptive coping and greater emotional stability, patterns that may correspond with constructive social engagement. Emotion regulation processes-particularly cognitive reappraisal-have also been linked to how people respond to emotionally challenging events. Reappraisal involves reinterpreting the meaning of a situation to alter its emotional impact, and studies have associated its use with reduced negative affect and improved interpersonal functioning.

Taken together, these lines of research suggest that reappraisal may account for part of the observed association between resilience and prosocial behavior. However, relatively few studies have directly examined this indirect pathway, especially among college students who frequently encounter academic and social stressors. To address this gap, the present study investigated whether cognitive reappraisal shows a statistical indirect association with the resilience–prosocial behavior link in a cross-sectional sample of Chinese college students.

### 1.1 Psychological resilience and prosocial behavior

Prosocial behavior is an important indicator of socialization levels, and a key factor in individual social adaptation. The theory of altruism in the face of suffering proposed by Staub (Huand Yang, 2022) suggests that resilient individuals are more likely to exhibit altruistic behavior when encountering adverse and traumatic experiences, as a way to reciprocate the help they have received from others (Uranus, 2022). Resilience, as an important positive personal resource, helps individual scope with traumatic events by enhancing self-efficacy, problem-solving skills, and adaptability, thereby promoting greater altruistic behavior.

Having strong psychological resilience is regarded as an essential resource for maintaining mental health in the face of major disasters and adversity. Research has found a correlation between psychological resilience and mental health, and enhancing an individual’s level of resilience can help promote their psychological wellbeing (Miao and Qiao, 2021). People with good mental health are typically better at regulating emotions and maintaining a positive mindset. This emotional regulation ability makes them more willing to help others, and they tend to exhibit a greater level of prosocial behavior. Positive emotions can enhance an individual’s empathy, motivating them to provide support to others (Quinn and Simmons, 2021).

Kumpfer (1999) developed a framework of psychological resilience that describes the interactions between risk factors and protective factors within the environmental context, where protective factors servea buffering function (Zhang and Flannery, 2023) Generally, individual scan adapt well when faced with one or two risk factors, but the probability of developmental dysfunction and maladaptation significantly increases when they are exposed to more than two risk factors. Conversely, an increase in the number of protective factors can effectively buffer the impact of these risk factors.

Psychological resilience is regarded as an important psychological resource that enables individuals to maintain mental health in the face of major stressors and adversity. Previous studies have demonstrated a close association between psychological resilience and mental health, indicating that higher levels of resilience help individuals regulate their emotions more effectively and maintain a positive mindset, thereby facilitating psychological adjustment and healthy development (Miao and Qiao, 2021). Adopting asocial-ecological and person–context interaction perspective, Kumpfer (1999) proposed a resilience framework that systematically explains the dynamic relationships among environmental risk factors and protective factors, individuals’ internal psychological characteristics, and person–environment interaction processes. This framework emphasizes that protective factors can buffer the negative effects of risk factors on individual development, and that individuals with strong psychological resilience are more likely to transform high-risk situations into relatively favorable environments through selective cognition, positive environmental restructuring, and proactive coping strategies. In addition, support and positive socialization from significant others in the family, school, and community provide critical foundations for the development and maintenance of psychological resilience.

Psychological resilience not only contributes to individual mental health, but may also play an important role in promoting prosocial behavior. Effective emotion regulation is a key component of psychological resilience, and positive emotional experiences can enhance individuals’ empathy and sense of social responsibility, thereby increasing their motivation and tendency to engage in helping behavior (Quinn and Simmons, 2021).

### 1.2 Psychological resilience and emotional reappraisal

Psychological resilience is widely recognized as a key factor in mitigating the detrimental effects of stress, with effective emotion regulation—particularly cognitive reappraisal—playing a central role in its development and maintenance. Emotional reappraisal refers to the process of cognitively reframing stressors to diminish negative emotional responses, a mechanism intricately linked to both the protective and promotive functions of psychological resilience. Models of emotion regulation posit that cognitive processes such as selective attention and evaluation can proactively modify affective experiences, thereby attenuating neural responses in regions such as the amygdala and insula (Messina and Viviani, 2021). Studies have demonstrated that individuals employing adaptive strategies —including university students and preschool children — recover more efficiently from adverse events and exhibit a reduced risk of compromised resilience (Gross and Zhang, 2020; Troy et al., 2023).

Moreover, the neural and cognitive mechanisms involved in cognitive reappraisal are associated with enhancements in metacognitive functioning and coping abilities, which further rein force psychological resilience and foster prosocial behavior. Research indicates that individuals capable of reinterpreting negative life events in a more objective manner not only experience diminished psychological burden but also facilitate more effective social interactions and adaptive coping strategies (Rodman, 2019; Lincoln and Renneberg, 2022). The dual benefits of cognitive reappraisal in modulating emotional experience and promoting social engagement underscore its crucial mediating role in the dynamic interplay between stress and resilience.

In summary, by altering how individuals interpret adverse events, cognitive reappraisal appears to relate to reduced negative affect and enhanced psychological adjustment. This regulatory strategy may also contribute to improved social adaptation, offering a potential route through which resilience and prosocial behavior are statistically associated in cross-sectional data.

### 1.3 Psychological resilience, prosocial behavior, and emotional reappraisal

Psychological resilience is closely linked to positive emotions. Research shows that individuals with high resilience can regulate their emotions more effectively and recover faster from negative feelings when facing adverse events (Maggalinggam and Ramlee, 2021). A study of 207 Chinese college students found that positive emotions predicted resilience 9 weeks later (Li et al., 2021). The present study examines the mediating effect of cognitive reappraisal on the relationship between positive emotions and resilience. Gross’s process model of emotion regulation underscores the central role of cognitive change in enhancing positive emotional experiences. Specifically, cognitive reappraisal intervenes before an emotional response is fully formed, thereby dampening negative emotional intensity. Supporting this view, neurobiological studies have shown that employing cognitive reappraisal to manage negative emotions is associated with reduced activity in emotion-related brain regions such as the amygdala and insula (Messina and Viviani, 2021).

In probing the role of cognitive reappraisal, Kumpfer’ s psychological resilience model posits cognition as a critical internal factor influencing resilience (Daniels, 2023). Building on this, Mauss and Troy (2024) integrated past research to illustrate the profound impact of reappraisal on resilience, proposing that cognitive reappraisal mediates the link between adverse events and psychological resilience. Although scholars have variously defined resilience, its positive impact on mental health is widely acknowledged, as it enables individuals to recover better from adverse life events and mitigate negative psychological outcomes. Effective cognitive reappraisal helps maintain a positive emotional state and serves as a protective factor that fosters resilience. Numerous studies have documented a close association between cognitive reappraisal and resilience. For example, research on preschoolers (Zhang, 2020) found that higher reappraisal ability mediates the relationship between risk factors and lower levels of resilience, while Rodman (2019) reported that neural circuits involved in cognitive control of emotion protect resilience by facilitating reappraisal of negative experiences, ultimately reducing adverse impacts on children’s psychology.

Furthermore, cultivating positive and effective coping strategies may facilitate the development of prosocial behavior among college students. Personality is an important determinant of prosocial tendencies, yet there is no consensus on how stable personality traits related to resilience affect prosocial behavior. As a positive coping resource, resilience effectively buffers the negative impact of stress (Sun, 2021). For instance, Wang (2019) studied undergraduate nursing students and found that various dimensions of resilience such as optimism, problem-solving ability, and social skills were significantly correlated with prosocial behavior. When scores of optimism, problem-solving ability, social interaction skills, and self-efficacy were used as predictors, and prosocial behavior as the outcome, self-efficacy indirectly influenced prosocial behavior through the process of cognitive reappraisal. However, research has yet to explore the mediating role of reappraisal in the relationship between resilience and prosocial behavior. Strengthening resilience can improve individual adaptability and reduce anxiety risk. Coping strategies, which are cognitive or behavioral responses to demands exceeding individual resources, play a critical role in buffering stress and maintaining wellbeing (Bondarchuk et al., 2024), with effective coping being essential for enhanced resilience (Feyisa et al., 2022).

Psychological resilience refers to the ability to maintain internal balance and adapt to challenging environments, primarily by effectively managing and regulating emotions to alleviate the adverse effects of negative feelings. Recent studies have highlighted the critical role of cognitive reappraisal, a positive emotion regulation strategy, in promoting resilience, and called for examinations of its mediating effect in the process of regulating and recovering positive emotional experiences. For example, Troy (2023) found that anticipatory cognitive processing of upcoming emotions can significantly reduce negative emotional intensity, illustrating the mediating role of reappraisal and offering a novel metacognitive perspective that encourages constructive coping in adversity. Similarly, (White et al., 2023) demonstrated that repetitive use of cognitive reappraisal not only strengthens adaptive coping mechanisms but also facilitates a quicker recovery of positive emotions under stress.

Moreover, the interplay between psychological resilience and cognitive reappraisal has significant theoretical implications for fostering prosocial behavior. Prosocial behaviors characterized by care, cooperation, and a willingness to help are closely linked to how individuals manage stress and adversity. A strong capacity for reappraisal enables individuals to maintain emotional balance under pressure, reducing psychological burdens and stimulating the intrinsic motivation needed for positive social interactions. Recent research suggests that individuals with both high resilience and effective reappraisal skills tend to exhibit greater empathy and responsibility (Troy et al., 2023; White et al., 2023), a relationship that warrants further investigation particularly research that assesses the mediating role of cognitive reappraisal. The evidence cited above suggests that incorporating reappraisal techniques into mental health interventions might simultaneously bolster resilience and encourage prosocial behavior.

In sum, the literature suggests that resilience, reappraisal, and prosocial behavior are positively interrelated. Individuals who report higher resilience tend to use reappraisal more frequently, and more frequent use of reappraisal is associated with higher levels of prosocial behavior. However, the precise nature of these interrelations remains unclear. Most existing studies have examined these constructs separately or in pairwise combinations, and relatively few have assessed whether reappraisal accounts for part of the resilience–prosocial behavior association within the same sample. The present study addresses this gap by examining the statistical dependencies among the three constructs in a cross-sectional sample of Chinese college students. Specifically, we investigated whether cognitive reappraisal shows a statistical indirect association with the resilience–prosocial behavior link.

### 1.4 The present study

Although prior research has established independent associations among psychological resilience, cognitive reappraisal, and prosocial behavior, the mechanisms underlying their interrelationships remain insufficiently understood. Psychological resilience, as an individual’ s capacity to adapt to stress and adversity, has been consistently linked to positive social functioning (Szabó et al., 2020). At the same time, cognitive reappraisal- an adaptive emotion regulation strategy involving the reinterpretation of emotional stimuli has been shown to play a key role in shaping both emotional experiences and social behaviors (Wang and Yin, 2023).

From a theoretical perspective, integrating psychological resilience with cognitive reappraisal may provide a more comprehensive understanding of how internal coping resources translate into observable social outcomes. In particular, individuals with higher levels of resilience may be more likely to engage in adaptive cognitive regulation strategies, such as reappraisal, which in turn facilitate constructive social interactions. Furthermore, specific dimensions of resilience, such as toughness and self-reliance, may differentially relate to the use of reappraisal, highlighting the need for a more nuanced conceptualization of resilience and its functional pathways.

Recent neurocognitive and psychological research has emphasized the dynamic role of cognitive reappraisal in modulating negative affect and supporting positive interpersonal engagement (Yu et al., 2025). This suggests that reappraisal may serve as an important link between resilience-related cognitive processes and prosocial behavioral tendencies. Yet direct empirical tests of this possibility, particularly in college student samples, remain relatively scarce.

Based on this theoretical and empirical background, the present study aims to examine the relationships among psychological resilience, cognitive reappraisal, and prosocial behavior, with a particular focus on the mediating role of cognitive reappraisal. Accordingly, the following hypotheses are proposed:

*H_*a*_1*: Psychological resilience is significantly positively correlated with prosocial behavior.

*H_*a*_2*: Psychological resilience is significantly positively correlated with cognitive reappraisal.

*H_*a*_3*: Cognitive reappraisal plays a statistical mediating role between psychological resilience and prosocial behavior.

## 2 Materials and methods

### 2.1 Research design

Prior to formal analysis, the dataset underwent systematic screening to verify its suitability for statistical testing. Cases exceeding the threshold for missing responses were excluded; for the remaining observations, missing values were addressed using expectation-maximization imputation. Outliers were identified through a combination of Mahalanobis distance and standardized scores, and decisions regarding their retention or removal were based on plausibility and their potential influence on parameter estimates. Duplicate entries, once detected through cross-comparison of demographic and response patterns, were deleted. In addition, all variables were checked for consistency in coding, range restrictions, and formatting accuracy.

Given that all measures were administered via self-report, the potential threat of common method bias was examined using Harman’s single-factor test. All scale items were entered into an unrotated exploratory factor analysis. The first extracted factor accounted for 30.96% of the total variance, falling below the conventional threshold of 40%, suggesting that common method variance did not pose a substantial concern in this study.

Following data cleaning, descriptive statistics—including means, standard deviations, medians, and ranges—were computed for the core variables to characterize the sample’s distribution on psychological resilience, prosocial behavior, and cognitive reappraisal. Bivariate associations among these variables were then examined using Pearson product-moment correlations, with partial correlations reported after controlling for gender, academic year, and only-child status.

The test of the indirect effect was conducted in accordance with contemporary recommendations for mediation analysis (Hayes et al., 2018; Hayes, 2022), using ordinary least squares regression coupled with bootstrapping procedures. Specifically, we employed the PROCESS macro for SPSS (Model 4; Hayes et al., 2018) to estimate the indirect effect and its associated bias-corrected 95% confidence interval, based on 5,000 bootstrap resamples. Statistical significance of the indirect effect was determined by whether the bootstrap confidence interval excluded zero. This approach does not require all individual paths to reach statistical significance and offers improved statistical power and more accurate Type I error control relative to the traditional causal-steps framework (Hayes, 2022; MacKinnon et al., 2004). In reporting the results, we present the indirect effect estimate, its bootstrapped standard error, the confidence interval, and the proportion of the total effect accounted for by the indirect pathway.

Because all variables were measured concurrently, the cross-sectional nature of the data precludes directional or causal inferences (Maxwell and Cole, 2007; Kline, 2016). To empirically illustrate this limitation, we tested two alternative models that are statistically equivalent or near-equivalent to the proposed model given the observed covariance structure: (a) a reverse mediation model, in which cognitive reappraisal predicts psychological resilience, which in turn predicts prosocial behavior; and (b) a common-cause model, in which an unobserved third variable accounts for the shared variance among the three constructs. By comparing model fit indices and indirect effect estimates across these competing specifications, we explicitly demonstrate that multiple directional interpretations are compatible with the same covariance matrix (MacKinnon, 2008; Kline, 2016). All models fit were estimated using AMOS.

### 2.2 Participants

A Monte Carlo power analysis for mediation models was conducted following the recommendations of Schoemann et al. (2017) to determine the required sample size for detecting the hypothesized indirect effect. The power analysis was performed using the authors’ R-based application, which implements Monte Carlo simulations to estimate statistical power for indirect effects tested via Monte Carlo confidence intervals.

Based on prior studies in similar research contexts, we set the path coefficients as follows: *a* = 0.30 (predictor to mediator), *b* = 0.30 (mediator to outcome), and direct effect c’ = 0.10, yielding a small-to-moderate indirect effect (ab = 0.09). The simulation used 5,000 replications and 20,000 draws per replication to generate the sampling distribution. We targeted a statistical power of 0.80 with αα = 0.05 (two-tailed).

The results showed that approximately 310 participants were needed to achieve 80% power for detecting the indirect effect. To be conservative and account for possible data loss, we oversampled using a stratified cluster strategy. Specifically, we selected one university from each of the eight prefecture-level cities in Guizhou Province. In total, 430 questionnaires were distributed. After careful data cleaning, 411 valid responses were retained (response rate = 95.60%). This final sample size (*N* = 411) exceeds the recommended minimum of 310, suggesting the study has adequate power to test the proposed mediation model.

As shown in Table 1, 275 were male (66.90%) and 136 female (33.10%). Forty-eight participants (11.67%) were only children, and 363 (88.33%) had siblings. Class cadres accounted for 117 participants (28.46%), while 294 (71.54%) were not. Ages ranged from 18 to 25 years (*M* = 22.14, SD = 2.55).

**TABLE 1:** Distribution of main demographic variables *n* = 411.

Variable	Category	Number	Percentage
Gender	Male	275	66.90%
	Female	136	33. 10%
Only child	Yes	48	11.67%
	No	363	88.33%
Class cadres	Yes	117	28.46%
	No	294	71.54%

Participants were administered three survey questionnaires:

Prosocial Tendencies Measure (PTM): The Chinese version of the Prosocial Tendencies Measure (PTM), which has been localized and adjusted for assessing the prosocial behavior of Chinese college students (Fu et al., 2018), was used. It comprises 25 items grouped into six factors: Compliance, Public, Altruistic, Fearful, Emotional, and Anonymous. Responses to each item are scored on a 5-point Likert scale ranging from 1 (strongly disagree) to 5 (strongly agree),with higher scores indicating higher levels of prosocial behavior. The Cronbach’s α coefficients for the dimensions and the overall scale range from 0.75 to 0.82, and the test-retest reliability ranges from 0.84 to 0.92, indicating good content validity and reliability appropriate for assessment in Chinese college students.

Based on the Table 2, a reliability and validity analysis was conducted on the PTM scale. The fit indices for this scale were as follows: *χ^2^*/df = 3.438, RMSEA = 0.077, NFI = 0.835, RFI = 0.819, IFI = 0.877, TLI = 0.864, and CFI = 0.876. According to the fit index evaluation criteria proposed by Moss (2019), in which CFI values between 0.80 and 0.89 are considered acceptable fit and RMSEA values between 0.05 and 0.10 are considered acceptable fit, the majority of the fit indices for the PTM model in the present study fell within the acceptable range, with the CFI (0.876) and RMSEA (0.077) meeting these criteria. Nevertheless, it should be noted that some indices (e.g., NFI = 0.835, TLI = 0.864) did not reach the more stringent thresholds (e.g., CFI ≥ 0.90) suggested by Hu and Bentler (1998). Taken together, the model fit of the PTM scale can be considered marginal to acceptable in the context of this study.

**TABLE 2:** PTM model fit.

	χ^2^ */df*	NFI	RFI	IFI	TLI	CFI	RMSEA
PTM	3.438	0.835	0.819	0.877	0.864	0.876	0.077

Confirmatory factor analysis was conducted to evaluate the factor structure of the CD-RISC in the present sample. As show in the Table 3, fit indices yielded the following results: χ^2^/df = 2.737, RMSEA = 0.065, CFI = 0.896, NFI = 0.846, IFI = 0.897, and TLI = 0.885. The χ^2^/df ratio fell below the recommended threshold of 3, and the RMSEA value was under 0.08, both suggesting acceptable model fit. However, the CFI (0.896) and TLI (0.885) did not reach the conventional cutoff of 0.90 (Hu and Bentler, 1998), and the NFI (0.846) and IFI (0.897) also fell short of ideal standards. Taken together, these indices indicate that the model fit for the CD-RISC in this sample was marginal to acceptable. This finding is consistent with prior research documenting variability in the factor structure of the CD-RISC across different samples (Yu et al., 2011), and may partly reflect challenges associated with cross-cultural adaptation and dimensional instability when the scale is applied in the Chinese context.

**TABLE 3:** CD-RISC model fit.

	χ^2^ */df*	NFI	RFI	IFI	TLI	CFI	RMSEA
CD-RISC	2.737	0.846	0.831	0.897	0.885	0.896	0.065

Emotion Regulation Questionnaire (ERQ): The Emotion Regulation Questionnaire (ERQ), developed by Gross and John, was adapted for local use by Chinese psychology researchers Gong et al. (2022). The scale comprises 10 items divided into two dimensions: cognitive reappraisal and expressive suppression, scored on a 5-point scale, and with 7 items assessing cognitive reappraisal strategies and 3 items assessing expressive suppression strategies. Each item is a statement typically on a 7-point Likert scale ranging from “never” (1 point) to “always” (7 points). The Cronbach’s α coefficient for cognitive reappraisal was 0.80, and the corrected item–total correlation coefficients for the ERQ items ranged from 0.42 to 0.73.

As shown in the Table 4, the structural validity of the emotion regulation scale was assessed with the following fit indices: χ^2^/df = 2.737, CMIN = 86.988, RMSEA = 0.062, NFI = 0.946, RFI = 0.928, IFI = 0.966, TLI = 0.955, and CFI = 0.966. These metrics indicate that the scale exhibits excellent structural validity.

**TABLE 4:** ERQ model fit.

	χ^2^ */df*	NFI	RFI	IFI	TLI	CFI	RMSEA
ERQ	2.737	0.946	0.928	0.966	0.955	0.966	0.062

The study obtained an ethical clearance from the Human Research Ethics Committee of the Universiti Malaysia Sarawak (protocol number: HREC(NM)/2023 (2)/52). All participants voluntarily participated in the experiment and signed informed consent and confidentiality agreements. Participants were instructed to engage sincerely to ensure the authenticity of their responses, with the option to complete the questionnaires in three sessions if needed. After the researchers collected the completed questionnaires, participants were informed about the confidentiality of the research and measures taken to mitigate any negative impact on them.

## 3 Results

### 3.1 Common method bias test

Given that the data were collected via self-report questionnaires, there was a potential risk of common method bias (CMB). To examine whether common method variance posed a serious threat, Harman’s single-factor test was conducted. All items from the PTM scale, the CD-RISC, and the Cognitive Reappraisal scale were entered into an exploratory factor analysis (EFA) model. The results indicated in the Table 5, the first unrotated factor accounted for 30.96% of the total variance, which is below the commonly accepted threshold of 40%. Therefore, no significant common method bias was detected in the current sample, providing a methodological foundation for subsequent statistical inference.

**TABLE 5:** Results of Harman’s single-factor test for common method bias (total variance explained).

	Initial eigen values			Extracting the sum of squared loads			Sum of squares of rotational loads		
Element	Total	Percentage of variance	Accumulation%	Total	Percentage of variance	Accumulation%	Total	Percentage of variance	Accumulation%
1	17.65	30.96	30.96	17.65	30.96	30.96	8.88	15.57	15.57
2	5.89	10.33	41.29	5.89	10.33	41.29	6.36	11.17	26.74
3	2.30	4.04	45.33	2.30	4.04	45.33	4.84	8.50	35.24
4	1.93	3.39	48.72	1.93	3.39	48.72	3.82	6.71	41.94
5	1.46	2.57	51.28	1.46	2.57	51.28	3.64	6.38	48.33
6	1.43	2.51	53.79	1.43	2.51	53.79	2.45	4.29	52.62
7	1.23	2.15	55.94	1.23	2.15	55.94	1.66	2.92	55.53
8	1.13	1.99	57.93	1.13	1.99	57.93	1.37	2.40	57.93
9	0.99	1.74	59.67						
10	0.98	1.72	61.39						
11	0.93	1.63	63.02						
…									

#### 3.1.1 Discriminant validity

Discriminant validity between psychological resilience (CD-RISC) and cognitive reappraisal was assessed using the Fornell–Larcker criterion (Fornell and Larcker, 1981). As presented in Table 6, the average variance extracted (AVE) for CD-RISC was 0.678, and that for cognitive reappraisal was 0.721, yielding square roots of 0.823 and 0.849, respectively. The Pearson correlation coefficient between the two constructs was 0.580. According to the Fornell–Larcker criterion, discriminant validity is established when the square root of each construct’s AVE exceeds its correlation with any other construct. In the present study, both AVE square roots (0.823 and 0.849) were greater than the inter-construct correlation (0.580). These results support adequate discriminant validity between psychological resilience and cognitive reappraisal, indicating that the two constructs are empirically distinct despite being positively correlated.

**TABLE 6:** Discriminant validity: Fornell–Larcker criterion.

Construct	CD-RISC	Cognitive reappraisal
CD-RISC	**0.823**	
Cognitive reappraisal	0.580	**0.849**

Diagonal elements (in bold) represent the square roots of the average variance extracted (AVE). Off-diagonal elements are Pearson correlation coefficients between constructs. Discriminant validity is supported when diagonal values exceed the corresponding off-diagonal correlations. The HTMT value between the two constructs was 0.830, below the recommended threshold of 0.85.

To further corroborate this conclusion, the heterotrait–monotrait ratio of correlations (HTMT; Henseler et al., 2015) was calculated. The HTMT value between the two constructs was 0.830, which falls below the conservative threshold of 0.85 and the more lenient threshold of 0.90. This result provides additional support for adequate discriminant validity.

### 3.2 Descriptive analyses

Below is a preliminary exploration of the overall sample characteristics and the distribution of the core variables. Data were collected from 411 university students. Psychological resilience served as the independent variable, reappraisal was examined as the mediating variable, and prosocial behavior served as the dependent variable.

The results indicated in Table 7 the mean score for psychological resilience was 3.358 (SD = 0.719), suggesting that participants generally exhibited relatively high resilience. For cognitive reappraisal, the mean score was 3.657 (SD = 0.911), implying that most participants tend to adopt cognitive restructuring strategies to regulate their emotions. The prosocial behavior score yielded a mean of 3.923 (SD = 0.682), reflecting moderate prosocial tendencies. These statistics provide a solid foundation for the subsequent mediation analysis.

**TABLE 7:** Basic indicator sort.

Item	*n*	Min.	Max.	Mean	SD	Median
PTM-R	411	1.000	5.000	3.824	0.682	3.923
CD-RISC	411	1.000	5.000	3.358	0.719	3.240
Cognitive reappraisal	411	1.000	5.857	3.792	0.911	3.657

### 3.3 Correlation analysis of variables

The correlation analysis results indicate that prosocial behavior is positively correlated with active engagement, appropriate refusal, self-disclosure, conflict management, emotional support, and cognitive reappraisal strategies. Similarly, psychological resilience shows positive correlations with active engagement, appropriate refusal, self-disclosure, conflict management, emotional support, and cognitive reappraisal strategies. Additionally, cognitive reappraisal strategies are positively correlated with active engagement, appropriate refusal, self-disclosure, conflict management, and emotional support. The detailed results are presented in Table 8.

**TABLE 8:** Partial correlation analysis results among key variables (controlling for gender, academic year, and only-child status).

	1	2	3
1. Psychological resilience	1	1	1
2. Prosocial behavior	0.469***		
3. Emotion reappraisal	0.587***	0.474***	

*P < 0.05, **P < 0.01, ***P < 0.001.

After controlling for gender, academic year, and only-child status, a correlation analysis among the four main variables revealed the following findings. First, psychological resilience was found to be significantly positively correlated with prosocial behavior (*r* = 0.469, *p* < 0.001), indicating a moderately high positive association. This suggests that, after accounting for gender, academic year, and only-child status, individuals with higher levels of psychological resilience tend to exhibit more pronounced prosocial behaviors. Second, a significant positive correlation was found between psychological resilience and the cognitive reappraisal dimension (*r* = 0.587, *p* < 0.001), representing a moderately strong association. This indicates that individuals with higher psychological resilience are more inclined to employ cognitive reappraisal strategies. Third, prosocial behavior showed a significant positive correlation with the cognitive reappraisal dimension (*r* = 0.474, *p* < 0.001), suggesting a moderately high positive relationship after controlling for the other variables. These results underscore the interconnected nature of the examined constructs in the sample, with statistically significant associations across all relationships described.

### 3.4 Regression analysis of variables

From the Table 9 In a sample of 411 college students, psychological resilience positively predicts prosocial behavior. A simple linear regression was performed with prosocial behavior (PTM-R) as the dependent variable and psychological resilience (CD-RISC) as the predictor. The unstandardized coefficient for CD-RISC was 0.490 [*t*(409) = 10.732, 95% CI = 0.400–0.580], *p* < 0.001. The model explains 22.0% of the variance in prosocial behavior (*R*^2^ = 0.220; adjusted *R*^2^ = 0.218). The intercept is 57.645 [*t*(14.988) =14.988, *p* < 0.001]. The overall model is significant [*F*(1, 409) = 115.175, *p* < 0.001]. Collinearity diagnostics show no evidence of multicollinearity (VIF =1.000, Tolerance =1.000). These findings support Ha 1, higher psychological resilience is associated with higher prosocial behavior among college students.

**TABLE 9:** Simple linear regression results of psychological resilience on prosocial behavior.

	Coef.	95% CI	Collinearity diagnostic	
			VIF	tolerance
Constant	57.645** (14.988)	50.085 ∼65.205	-	-
CD-RISC	0.490** (10.732)	0.400 ∼ 0.580	1.000	1.000
*n*	411			
R-squared	0.220			
Adjust R-squared	0.2183			
*F-*Test	*F*(1, 409) = 115.175, *p* = 0.000			

Dependent Variable = PTM-R D-W = 1.983. *p < 0.05 **p < 0.01, *t*-value in parentheses.

As shown in Table 10, a regression equation was estimated with psychological resilience as the predictor and emotional reappraisal as the outcome variable. The unstandardized coefficient for psychological resilience was 0.202 (*t* = 14.659, *p* < 0.01), with a 95% confidence interval of [0.175, 0.229] that did not include zero. The model yielded an *R*^2^ of 0.344 and an adjusted *R*^2^ of 0.343, indicating that psychological resilience explained approximately 34.4% of the variance in emotional reappraisal. The overall model was significant, *F*(1, 409) = 214.887, *p* < 0.001. Collinearity diagnostics showed a variance inflation factor of 1.000 and tolerance of 1.000, suggesting no multicollinearity concerns. The Durbin-Watson statistic was 1.850, close to 2, indicating a low likelihood of residual autocorrelation. Taken together, these results point to a moderate positive association between psychological resilience and emotional reappraisal.

**TABLE 10:** Simple linear regression results of psychological resilience on emotional reappraisal.

	Coef.	95% CI	Collinearity diagnostic	
			VIF	tolerance
Constant	0.585** (4.084)	0.304 ∼ 0.867	-	-
CD-RISC	0.202** (14.659)	0.175 ∼ 0.229	1.000	1.000
*n*	411			
R-squared	0.344			
Adjust R-squared	0.343			
*F-*test	*F*(1, 409) = 214.887, *p* = 0.000			

Dependent Variable = Emotional reappraisal D-W = 1.850. *p < 0.05 **p < 0.01, *t*-value in parentheses.

### 3.5 The mediating role of emotion reappraisal strategies in the relationship between psychological resilience and prosocial behavior

This study examined the indirect role of cognitive reappraisal in the relationship between psychological resilience and prosocial behavior using cross-sectional data. Following contemporary best practices for mediation analysis (Hayes et al., 2018; Hayes, 2022), the indirect effect was estimated using ordinary least squares regression with bootstrapping procedures. Specifically, we employed the PROCESS macro for SPSS (Model 4; Hayes et al., 2018) to estimate the indirect effect and its associated bias-corrected 95% confidence interval based on 5,000 bootstrap resamples. Statistical significance of the indirect effect is determined by whether the bootstrap confidence interval excludes zero, a method that does not require all individual paths to be statistically significant and provides superior statistical power relative to the causal steps approach (Hayes, 2022).

**FIGURE 1:**
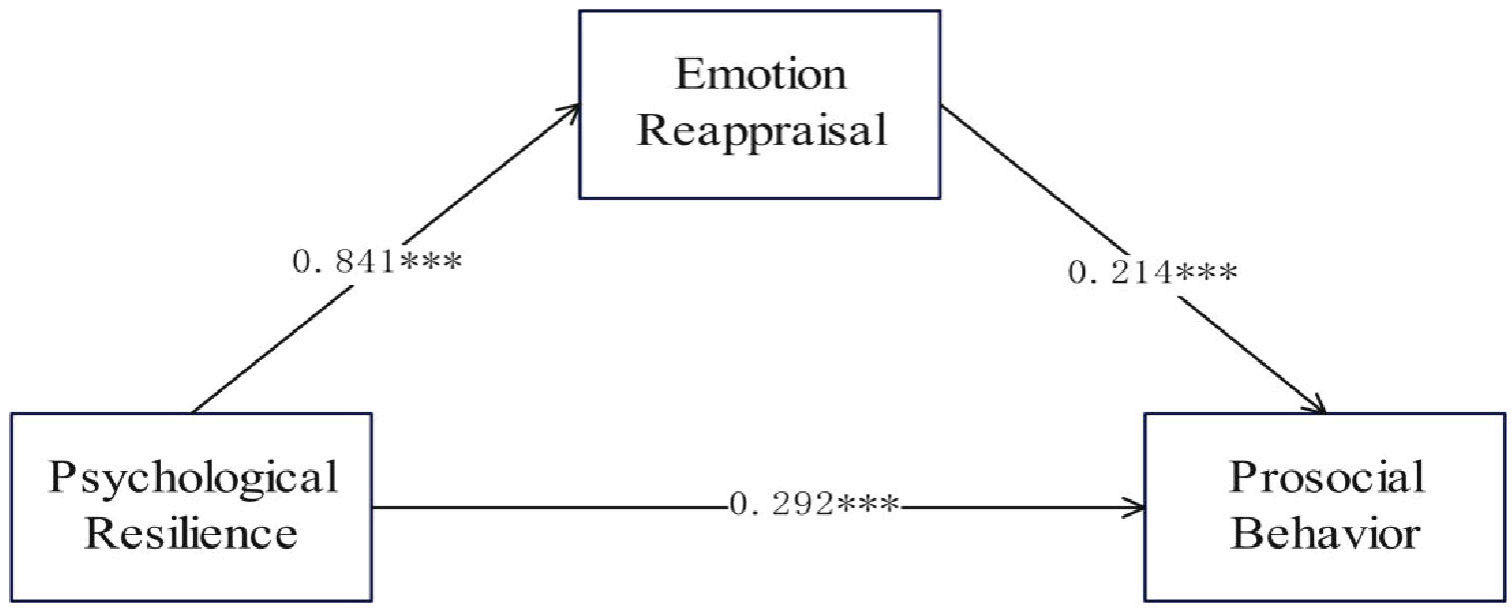
Mediation effects of emotion reappraisal in the relationship between psychological resilience and prosocial behavior.

As shown in Table 11 and Figure 1, psychological resilience demonstrated a significant bivariate association with prosocial behavior (β = 0.471, *R*^2^ = 0.220, *p* < 0.001). Psychological resilience was also positively associated with emotion reappraisal (β = 0.841, *R*^2^ = 0.344, *p* < 0.001). When both psychological resilience and emotion reappraisal were entered simultaneously as predictors of prosocial behavior, both coefficients remained significant: emotion reappraisal (β = 0.214, *t* = 5.862, *p* < 0.001) and psychological resilience (β = 0.292, *t* = 5.596, *p* < 0.001). This pattern indicates that the association between psychological resilience and prosocial behavior is partially shared with emotion reappraisal, although the direct association remains robust.

**TABLE 11:** Regression coefficients for the indirect effect model.

Outcome variable	Predictor variable	*R*	*R* ^2^	F	β	*t*	*p*
Prosocial behavior	Psychological resilience	0.469	0.220	115.175	0.471	10.732	< 0.001
Cognitive reappraisal	Psychological resilience	0.587	0.344	214.887	0.841	14.659	< 0.001
Prosocial behavior	Cognitive reappraisal	0.530	0.280	79.469	0.214	5.862	< 0.001
	Psychological resilience				0.292	5.596	< 0.001

All coefficients are unstandardized (β) unless otherwise noted **P < 0.001.

Table 12 presents the decomposition of effects. The total association between psychological resilience and prosocial behavior was estimated at 0.471 [BootSE = 0.044; 95% CI (0.385, 0.558)]. The direct effect, representing the association between psychological resilience and prosocial behavior after accounting for emotion reappraisal, was 0.292 [BootSE = 0.052; 95% CI (0.189, 0.395)]. Critically, the indirect effect of psychological resilience on prosocial behavior through emotion reappraisal was estimated at 0.180 [BootSE = 0.041; 95% CI (0.100, 0.263)]. Because the bootstrap confidence interval excludes zero, the indirect effect is statistically significant at *p* < 0.001. This indirect effect accounts for approximately 38.2% of the total effect, indicating a meaningful indirect pathway.

**TABLE 12:** Decomposition of total, direct, and indirect effects.

Effect	Estimate	BootSE	95% BootLLCI	95% BootULCI	Proportion of total
Total effect	0.471***	0.044	0.385	0.558	–
Direct effect	0.292***	0.052	0.189	0.395	62.0%
Indirect effect via emotion reappraisal	0.180*	0.041	0.100	0.263	38.2%

*Note: ***p < 0.001. BootSE, bootstrapped standard error; LLCI, lower level 95% confidence interval; ULCI, upper level 95% confidence interval. Bootstrapping based on 5,000 resamples. Proportion of total effect accounted for by the indirect effect is calculated as indirect effect/total effect.

As show in the Table 13, the structural model demonstrated excellent fit to the data. The chi-square ratio (χ^2^/df = 1.777) fell well below the conventional threshold of 2. Incremental fit indices NFI = 0.985, RFI = 0.974, IFI = 0.993, TLI = 0.988, and CFI = 0.993-all exceeded the 0.95 benchmark. The RMSEA of 0.044 was below the 0.05 cutoff. These indices collectively indicate that the hypothesized model structure is consistent with the observed covariance pattern in the data.

**TABLE 13:** Goodness-of-fit indices for the mediation model.

	χ ^2^ */df*	NFI	RFI	IFI	TLI	CFI	RMSEA
Psychological resilience → emotion reappraisal → prosocial behavior	1.777	0.985	0.974	0.993	0.988	0.993	0.044

Default model: χ^2^(6) =711.769, *p* =1.000 AI = 25.836, BIC = 78.078.

### 3.6 Comparison of competing models

To acknowledge the inherent limitations of cross-sectional data in establishing directional pathways, we tested two alternative models that are statistically equivalent or near-equivalent to the proposed model given the concurrent measurement design (MacKinnon, 2008; Kline, 2016). Table 14 presents a comparison of the three competing models.

**TABLE 14:** Comparison of competing models.

Model	Directional path	Indirect effect estimate	95% BootCI	Model fit (CFI)	Model fit (RMSEA)
Model a (Proposed)	Resilience → reappraisal → prosocial Behavior	0.180	[0.100, 0.263]	0.993	0.044
Model b (reverse)	Reappraisal → resilience → prosocial behavior	0.183	[0.101, 0.269]	0.993	0.044
Model c (common-cause)	Unobserved third variable → all observed variables			0.993	0.044

All models are statistically equivalent or near-equivalent in terms of fit indices given the cross-sectional nature of the data. Bootstrapping based on 5,000 resamples.

As shown in Table 14, the reverse mediation model (Model b: Cognitive Reappraisal → Psychological Resilience → Prosocial Behavior) yielded an indirect effect estimate of 0.183 [BootSE = 0.043; 95% CI (0.101, 0.269)], which is nearly identical to that of the proposed model (0.180). This finding is not surprising given that the two models are statistically equivalent when variables are measured concurrently; the covariance structure does not distinguish between these directional alternatives (MacKinnon, 2008). Similarly, the common-cause model (Model c), in which an unobserved third variable accounts for the shared variance among resilience, reappraisal, and prosocial behavior, produced identical fit indices (CFI = 0.993; RMSEA = 0.044). These equivalent fits are a mathematical consequence of the cross-sectional design: all three models reproduce the observed covariance matrix equally well (Kline, 2016).

While the proposed model is consistent with the theoretical framework and received empirical support in terms of its indirect effect, the reverse and common-cause alternatives remain equally plausible given the concurrent measurement. Therefore, any claims regarding directional or causal ordering would be unjustified. The significant indirect effect reported in Model a.

## 4 Discussion

This study examined how psychological resilience, cognitive reappraisal, and prosocial behavior are related in a sample of Chinese college students. The results showed that psychological resilience was positively associated with prosocial behavior (β = 0.471, *p* < 0.001). Cognitive reappraisal accounted for a portion of this association, with an indirect effect of 0.180 [95% BootCI (0.100, 0.263)], representing about 38% of the total effect.

These findings align with earlier work suggesting that individuals who report higher resilience also tend to use adaptive emotion regulation strategies more frequently, and that this pattern relates to more favorable social outcomes (Tugade and Fredrickson, 2004; Troy et al., 2013). Cognitive reappraisal—reframing how one thinks about emotionally charged situations—appears to be one of the routes through which resilience and prosocial behavior are connected. This interpretation fits with existing research showing that reappraisal is consistently linked to better interpersonal functioning (Gross and John, 2004; English et al., 2014).

That said, the cross-sectional nature of the data places clear boundaries on what can be concluded. The indirect effect reported here is a statistical decomposition of concurrent covariation, not evidence that resilience precedes reappraisal or that reappraisal precedes prosocial behavior in time. Maxwell and Cole (2007) and Maxwell et al. (2011) have shown that cross-sectional estimates of indirect effects can deviate substantially from longitudinal estimates, and the direction of that deviation depends on factors—such as the true time lag between measurements—that cannot be recovered from a single wave of data.

To illustrate this point more concretely, we compared three competing models that are statistically equivalent given the cross-sectional design: (a) the proposed model (Resilience → Reappraisal → Prosocial Behavior), (b) a reverse-path model (Reappraisal → Resilience → Prosocial Behavior), and (c) a common-cause model in which an unobserved third variable accounts for the shared variance among all three constructs. All three models produced nearly identical fit indices (CFI = 0.993; RMSEA = 0.044), and the indirect effect for the reverse model [0.183, 95% CI (0.101, 0.269)] was practically the same as for the proposed model [0.180, 95% CI (0.100, 0.263)]. This equivalence is not a shortcoming of the analysis; it is a mathematical property of cross-sectional data. With concurrent measurements, the covariance structure alone cannot distinguish between competing directional explanations (MacKinnon, 2008; Kline, 2016). The reverse model is not theoretically implausible either—emotion regulation strategies may well shape resilience over time by helping individuals reframe adversities (Troy et al., 2013; Zautra, 2010). The current data simply cannot rule it out.

Looking at the direct and indirect components, both were statistically significant: the direct association between resilience and prosocial behavior [β = 0.292, *p* < 0.001) and the indirect association via cognitive reappraisal (β = 0.180, *p* < 0.001). The direct path accounted for 62% of the total effect, and the indirect path for 38%. A more straight forward interpretation is that resilience and prosocial behavior are related through multiple channels, and cognitive reappraisal is one of them. The measurement properties of the instruments warrant some comment. Confirmatory factor analyses showed that the fit indices for the Prosocial Tendencies Measure (CFI = 0.876, NFI = 0.835, RMSEA = 0.077) and the CD-RISC (CFI = 0.896, RMSEA = 0.065) fell below the commonly recommended threshold of 0.90 (Hu and Bentler, 1998), suggesting that the model fit was marginal to acceptable in the present sample. By contrast, the Emotion Regulation Questionnaire demonstrated comparatively better fit (CFI = 0.966, RMSEA = 0.062), which may be attributable to its fewer items and simpler factor structure. For the PTM and CD-RISC, the marginal fit may reflect challenges associated with cross-cultural adaptation (He and van de Vijver, 2012), as both instruments were originally developed in Western contexts and subsequently localized for Chinese samples.

In summary, the present findings are broadly consistent with the theoretical expectation that resilience and prosocial behavior are positively linked, and that cognitive reappraisal plays a role in that link. At the same time, the cross-sectional design and the equivalent fit of competing models preclude any strong conclusion about direction or causation. The measurement issues with the PTM and CD-RISC also suggest that results involving these instruments should be interpreted with some caution. What the study offers is a descriptive account of how these three constructs co-vary in a Chinese college sample, Whether cognitive reappraisal actually functions as a pathway in a temporal or causal sense remains an open question that future research-particularly longitudinal or experimental work will need to address.

### 4.1 Limitations and future research directions

Although this study has yielded some meaningful results, several limitations remain that future research should address. Cross-sectional design. The most important limitation is that all variables were measured at one time point. The mediation model used for analysis presumes a particular temporal ordering—resilience leading to reappraisal, and reappraisal leading to prosocial behavior—but the data do not provide direct evidence for that ordering. As noted earlier, alternative models with reversed or non-directional structures fit the data equally well. This means the indirect effect should be read as a statistical description of concurrent relationships, not as evidence of a causal chain. Future studies could address this by using longitudinal designs with at least three measurement occasions, which would allow for cross-lagged panel analysis or random-intercept cross-lagged models. Experimental designs that manipulate reappraisal use would offer a stronger basis for causal inference, though such designs come with their own practical and ethical considerations.

Sample and generalizability. The participants were 411 college students recruited from eight institutions in Guizhou Province, China. The sample size is adequate for the analyses conducted, but the findings may not extend to other populations. The gender distribution was skewed toward male participants (66.9%), and all participants were young adults in a specific regional and educational context. Whether similar patterns would emerge among older adults, non-students, or individuals from other cultural backgrounds remains an open question. Future research with more diverse samples—across age, education level, geographic region, and cultural setting—would help clarify the boundary conditions of the observed associations.

Self-report measures. All constructs were assessed through self-report questionnaires. This approach is efficient and widely used, but it carries known risks: socially desirable responding, inaccurate recall, and same-source variance that may inflate correlations. Although Harman’s test did not indicate serious common method bias, this test has limited sensitivity. Future work could strengthen the evidence base by incorporating other types of data—peer reports, behavioral observations, or physiological indicators—to see whether the relationships hold across different measurement methods. Mixed-method designs that combine surveys with qualitative interviews might also shed light on the processes underlying these associations.

### 4.2 Educational implications

The results confirmed significant positive associations among psychological resilience, cognitive reappraisal, and prosocial behavior. This pattern suggests that in college settings, these constructs may be interrelated rather than functioning independently. Students who reported higher levels of resilience tended to use reappraisal more often, and more frequent use of reappraisal was associated with higher levels of prosocial behavior.

Several directions for educational practice emerge from this pattern, though they remain tentative given the correlational design. One possibility is that mental health courses could include discussions of reappraisal and other emotion regulation strategies, helping students recognize everyday situations—academic setbacks or interpersonal conflicts, for instance—where reappraisal might be applied. Practical exercises could allow students to experience how different ways of interpreting an event shape their emotional and behavioral responses. In addition, because reappraisal is a describable and potentially teachable strategy, its indirect role in the resilience–prosocial behavior link suggests that fostering reappraisal skills could be one route through which internal psychological resources translate into socially oriented actions. Campus activities or peer support programs that involve perspective-taking and attention to others’ emotional experiences may also contribute to the development of both emotion regulation and resilience.

## Data Availability

The original contributions presented in the study are included in the article/Supplementary material, further inquiries can be directed to the corresponding author.
