# Supplementary material for: The indirect association between psychological resilience and prosocial behavior through cognitive reappraisal in Chinese college students
Source: Front Psychol. 2026 Sep 7;17:1789130. doi: 10.3389/fpsyg.2026.1789130 (PMC13591386; doi:10.3389/fpsyg.2026.1789130)
Supplement: Supplementary file 1 [file Data_Sheet_1.zip › 陈静数据分析/aoms数据/mmmm/mmmm.AmosOutput]

mmmm.amw


# C:\Users\86189\Desktop\mmmm.amw

## Analysis Summary

## Date and Time

Date: 2025年7月24日

Time: 1:01:21

## Title

mmmm: 2025年7月24日 1:01

## Groups

## Group number 1 (Group number 1)

## Notes for Group (Group number 1)

The model is recursive.

Sample size = 411

## Variable Summary (Group number 1)

## Your model contains the following variables (Group number 1)

Observed, endogenous variables

m5

m4

m6

m2

m1

m3

m8

m7

m10

m9

Unobserved, exogenous variables

F1

e1

e2

e3

e4

e5

e6

F2

e7

e8

e9

e10

## Variable counts (Group number 1)

|  |  |
| --- | --- |
| Number of variables in your model: | 22 |
| Number of observed variables: | 10 |
| Number of unobserved variables: | 12 |
| Number of exogenous variables: | 12 |
| Number of endogenous variables: | 10 |

## Parameter Summary (Group number 1)

|  | Weights | Covariances | Variances | Means | Intercepts | Total |
| --- | --- | --- | --- | --- | --- | --- |
| Fixed | 12 | 0 | 0 | 0 | 0 | 12 |
| Labeled | 0 | 0 | 0 | 0 | 0 | 0 |
| Unlabeled | 8 | 1 | 12 | 0 | 0 | 21 |
| Total | 20 | 1 | 12 | 0 | 0 | 33 |

## Models

## Default model (Default model)

## Notes for Model (Default model)

## Computation of degrees of freedom (Default model)

|  |  |
| --- | --- |
| Number of distinct sample moments: | 55 |
| Number of distinct parameters to be estimated: | 21 |
| Degrees of freedom (55 - 21): | 34 |

## Result (Default model)

Minimum was achieved

Chi-square = 86.988

Degrees of freedom = 34

Probability level = .000

## Group number 1 (Group number 1 - Default model)

## Estimates (Group number 1 - Default model)

## Scalar Estimates (Group number 1 - Default model)

## Maximum Likelihood Estimates

## Regression Weights: (Group number 1 - Default model)

|  |  |  | Estimate | S.E. | C.R. | P | Label |
| --- | --- | --- | --- | --- | --- | --- | --- |
| m5 | <--- | F1 | 1.000 |  |  |  |  |
| m4 | <--- | F1 | 1.060 | .068 | 15.497 | \*\*\* |  |
| m6 | <--- | F1 | .983 | .067 | 14.586 | \*\*\* |  |
| m2 | <--- | F1 | 1.057 | .073 | 14.427 | \*\*\* |  |
| m1 | <--- | F1 | .957 | .074 | 12.904 | \*\*\* |  |
| m3 | <--- | F1 | .856 | .070 | 12.229 | \*\*\* |  |
| m8 | <--- | F2 | 1.000 |  |  |  |  |
| m7 | <--- | F2 | 1.023 | .103 | 9.883 | \*\*\* |  |
| m10 | <--- | F2 | 1.259 | .112 | 11.219 | \*\*\* |  |
| m9 | <--- | F2 | 1.395 | .124 | 11.255 | \*\*\* |  |

## Covariances: (Group number 1 - Default model)

|  |  |  | Estimate | S.E. | C.R. | P | Label |
| --- | --- | --- | --- | --- | --- | --- | --- |
| F1 | <--> | F2 | .213 | .039 | 5.434 | \*\*\* |  |

## Variances: (Group number 1 - Default model)

|  |  |  | Estimate | S.E. | C.R. | P | Label |
| --- | --- | --- | --- | --- | --- | --- | --- |
| F1 |  |  | .637 | .075 | 8.534 | \*\*\* |  |
| F2 |  |  | .517 | .084 | 6.196 | \*\*\* |  |
| e1 |  |  | .479 | .042 | 11.503 | \*\*\* |  |
| e2 |  |  | .437 | .040 | 10.848 | \*\*\* |  |
| e3 |  |  | .501 | .043 | 11.725 | \*\*\* |  |
| e4 |  |  | .608 | .051 | 11.845 | \*\*\* |  |
| e5 |  |  | .754 | .059 | 12.695 | \*\*\* |  |
| e6 |  |  | .719 | .055 | 12.958 | \*\*\* |  |
| e7 |  |  | .873 | .071 | 12.373 | \*\*\* |  |
| e8 |  |  | .827 | .068 | 12.161 | \*\*\* |  |
| e9 |  |  | .537 | .059 | 9.059 | \*\*\* |  |
| e10 |  |  | .629 | .071 | 8.822 | \*\*\* |  |

## Minimization History (Default model)

| Iteration |  | Negative eigenvalues | Condition # | Smallest eigenvalue | Diameter | F | NTries | Ratio |
| --- | --- | --- | --- | --- | --- | --- | --- | --- |
| 0 | e | 4 |  | -.638 | 9999.000 | 1575.335 | 0 | 9999.000 |
| 1 | e | 2 |  | -.094 | 2.471 | 586.733 | 20 | .319 |
| 2 | e | 0 | 174.124 |  | 1.228 | 230.466 | 5 | .655 |
| 3 | e | 0 | 26.300 |  | .759 | 152.777 | 3 | .000 |
| 4 | e | 0 | 41.955 |  | .506 | 93.940 | 1 | 1.031 |
| 5 | e | 0 | 61.229 |  | .205 | 87.328 | 1 | 1.035 |
| 6 | e | 0 | 71.502 |  | .035 | 86.988 | 1 | 1.019 |
| 7 | e | 0 | 73.638 |  | .002 | 86.988 | 1 | 1.002 |
| 8 | e | 0 | 73.643 |  | .000 | 86.988 | 1 | 1.000 |

## Model Fit Summary

## CMIN

| Model | NPAR | CMIN | DF | P | CMIN/DF |
| --- | --- | --- | --- | --- | --- |
| Default model | 21 | 86.988 | 34 | .000 | 2.558 |
| Saturated model | 55 | .000 | 0 |  |  |
| Independence model | 10 | 1597.906 | 45 | .000 | 35.509 |

## RMR, GFI

| Model | RMR | GFI | AGFI | PGFI |
| --- | --- | --- | --- | --- |
| Default model | .052 | .960 | .935 | .593 |
| Saturated model | .000 | 1.000 |  |  |
| Independence model | .438 | .436 | .310 | .357 |

## Baseline Comparisons

| Model | NFI Delta1 | RFI rho1 | IFI Delta2 | TLI rho2 | CFI |
| --- | --- | --- | --- | --- | --- |
| Default model | .946 | .928 | .966 | .955 | .966 |
| Saturated model | 1.000 |  | 1.000 |  | 1.000 |
| Independence model | .000 | .000 | .000 | .000 | .000 |

## Parsimony-Adjusted Measures

| Model | PRATIO | PNFI | PCFI |
| --- | --- | --- | --- |
| Default model | .756 | .714 | .730 |
| Saturated model | .000 | .000 | .000 |
| Independence model | 1.000 | .000 | .000 |

## NCP

| Model | NCP | LO 90 | HI 90 |
| --- | --- | --- | --- |
| Default model | 52.988 | 29.220 | 84.433 |
| Saturated model | .000 | .000 | .000 |
| Independence model | 1552.906 | 1425.974 | 1687.210 |

## FMIN

| Model | FMIN | F0 | LO 90 | HI 90 |
| --- | --- | --- | --- | --- |
| Default model | .212 | .129 | .071 | .206 |
| Saturated model | .000 | .000 | .000 | .000 |
| Independence model | 3.897 | 3.788 | 3.478 | 4.115 |

## RMSEA

| Model | RMSEA | LO 90 | HI 90 | PCLOSE |
| --- | --- | --- | --- | --- |
| Default model | .062 | .046 | .078 | .109 |
| Independence model | .290 | .278 | .302 | .000 |

## AIC

| Model | AIC | BCC | BIC | CAIC |
| --- | --- | --- | --- | --- |
| Default model | 128.988 | 130.146 | 213.378 | 234.378 |
| Saturated model | 110.000 | 113.033 | 331.023 | 386.023 |
| Independence model | 1617.906 | 1618.458 | 1658.092 | 1668.092 |

## ECVI

| Model | ECVI | LO 90 | HI 90 | MECVI |
| --- | --- | --- | --- | --- |
| Default model | .315 | .257 | .391 | .317 |
| Saturated model | .268 | .268 | .268 | .276 |
| Independence model | 3.946 | 3.637 | 4.274 | 3.947 |

## HOELTER

| Model | HOELTER .05 | HOELTER .01 |
| --- | --- | --- |
| Default model | 230 | 265 |
| Independence model | 16 | 18 |

## Execution time summary

|  |  |
| --- | --- |
| Minimization: | .006 |
| Miscellaneous: | .101 |
| Bootstrap: | .000 |
| Total: | .107 |
