# Supplementary material for: The indirect association between psychological resilience and prosocial behavior through cognitive reappraisal in Chinese college students
Source: Front Psychol. 2026 Sep 7;17:1789130. doi: 10.3389/fpsyg.2026.1789130 (PMC13591386; doi:10.3389/fpsyg.2026.1789130)
Supplement: Supplementary file 1 [file Data_Sheet_1.zip › 陈静数据分析/aoms数据/model fit/2.AmosOutput]

2.amw


# C:\Users\86189\Desktop\2.amw

## Analysis Summary

## Date and Time

Date: 2025年7月24日

Time: 0:02:25

## Title

2: 2025年7月24日 0:02

## Groups

## Group number 1 (Group number 1)

## Notes for Group (Group number 1)

The model is recursive.

Sample size = 411

## Variable Summary (Group number 1)

## Your model contains the following variables (Group number 1)

Observed, endogenous variables

X3

X2

X1

Y3

Y2

Y1

Cognitivereappraisal

Unobserved, endogenous variables

PTM

Unobserved, exogenous variables

CD

E5

E4

E3

E8

E7

E6

E1

E2

## Variable counts (Group number 1)

|  |  |
| --- | --- |
| Number of variables in your model: | 17 |
| Number of observed variables: | 7 |
| Number of unobserved variables: | 10 |
| Number of exogenous variables: | 9 |
| Number of endogenous variables: | 8 |

## Parameter Summary (Group number 1)

|  | Weights | Covariances | Variances | Means | Intercepts | Total |
| --- | --- | --- | --- | --- | --- | --- |
| Fixed | 10 | 0 | 0 | 9 | 1 | 20 |
| Labeled | 0 | 0 | 0 | 0 | 0 | 0 |
| Unlabeled | 7 | 0 | 9 | 0 | 7 | 23 |
| Total | 17 | 0 | 9 | 9 | 8 | 43 |

## Models

## Default model (Default model)

## Notes for Model (Default model)

## Computation of degrees of freedom (Default model)

|  |  |
| --- | --- |
| Number of distinct sample moments: | 35 |
| Number of distinct parameters to be estimated: | 23 |
| Degrees of freedom (35 - 23): | 12 |

## Result (Default model)

Minimum was achieved

Chi-square = 21.320

Degrees of freedom = 12

Probability level = .046

## Group number 1 (Group number 1 - Default model)

## Estimates (Group number 1 - Default model)

## Scalar Estimates (Group number 1 - Default model)

## Maximum Likelihood Estimates

## Regression Weights: (Group number 1 - Default model)

|  |  |  | Estimate | S.E. | C.R. | P | Label |
| --- | --- | --- | --- | --- | --- | --- | --- |
| Cognitivereappraisal | <--- | CD | 17.702 | 3.807 | 4.650 | \*\*\* |  |
| PTM | <--- | Cognitivereappraisal | .030 | .007 | 4.560 | \*\*\* |  |
| PTM | <--- | CD | 1.133 | .303 | 3.735 | \*\*\* |  |
| X3 | <--- | CD | 1.000 |  |  |  |  |
| X2 | <--- | CD | 2.887 | .601 | 4.799 | \*\*\* |  |
| X1 | <--- | CD | 3.628 | .756 | 4.798 | \*\*\* |  |
| Y3 | <--- | PTM | 1.000 |  |  |  |  |
| Y2 | <--- | PTM | 1.084 | .050 | 21.533 | \*\*\* |  |
| Y1 | <--- | PTM | .928 | .047 | 19.670 | \*\*\* |  |

## Standardized Regression Weights: (Group number 1 - Default model)

|  |  |  | Estimate |
| --- | --- | --- | --- |
| Cognitivereappraisal | <--- | CD | .614 |
| PTM | <--- | Cognitivereappraisal | .271 |
| PTM | <--- | CD | .356 |
| X3 | <--- | CD | .245 |
| X2 | <--- | CD | .797 |
| X1 | <--- | CD | .947 |
| Y3 | <--- | PTM | .849 |
| Y2 | <--- | PTM | .895 |
| Y1 | <--- | PTM | .822 |

## Intercepts: (Group number 1 - Default model)

|  |  |  | Estimate | S.E. | C.R. | P | Label |
| --- | --- | --- | --- | --- | --- | --- | --- |
| Cognitivereappraisal |  |  | 23.630 | .251 | 94.235 | \*\*\* |  |
| X3 |  |  | 2.526 | .036 | 71.039 | \*\*\* |  |
| X2 |  |  | 3.458 | .032 | 109.754 | \*\*\* |  |
| X1 |  |  | 3.344 | .033 | 100.365 | \*\*\* |  |
| Y3 |  |  | 2.935 | .158 | 18.594 | \*\*\* |  |
| Y2 |  |  | 2.944 | .170 | 17.309 | \*\*\* |  |
| Y1 |  |  | 3.284 | .147 | 22.338 | \*\*\* |  |

## Variances: (Group number 1 - Default model)

|  |  |  | Estimate | S.E. | C.R. | P | Label |
| --- | --- | --- | --- | --- | --- | --- | --- |
| CD |  |  | .031 | .013 | 2.406 | .016 |  |
| E1 |  |  | 16.068 | 1.225 | 13.113 | \*\*\* |  |
| E2 |  |  | .214 | .022 | 9.840 | \*\*\* |  |
| E5 |  |  | .487 | .034 | 14.232 | \*\*\* |  |
| E4 |  |  | .149 | .016 | 9.349 | \*\*\* |  |
| E3 |  |  | .047 | .019 | 2.452 | .014 |  |
| E8 |  |  | .121 | .012 | 9.745 | \*\*\* |  |
| E7 |  |  | .092 | .012 | 7.453 | \*\*\* |  |
| E6 |  |  | .130 | .012 | 10.737 | \*\*\* |  |

## Matrices (Group number 1 - Default model)

## Total Effects (Group number 1 - Default model)

|  | CD | Cognitivereappraisal | PTM |
| --- | --- | --- | --- |
| Cognitivereappraisal | 17.702 | .000 | .000 |
| PTM | 1.662 | .030 | .000 |
| Y1 | 1.542 | .028 | .928 |
| Y2 | 1.802 | .032 | 1.084 |
| Y3 | 1.662 | .030 | 1.000 |
| X1 | 3.628 | .000 | .000 |
| X2 | 2.887 | .000 | .000 |
| X3 | 1.000 | .000 | .000 |

## Standardized Total Effects (Group number 1 - Default model)

|  | CD | Cognitivereappraisal | PTM |
| --- | --- | --- | --- |
| Cognitivereappraisal | .614 | .000 | .000 |
| PTM | .523 | .271 | .000 |
| Y1 | .430 | .223 | .822 |
| Y2 | .468 | .243 | .895 |
| Y3 | .444 | .230 | .849 |
| X1 | .947 | .000 | .000 |
| X2 | .797 | .000 | .000 |
| X3 | .245 | .000 | .000 |

## Direct Effects (Group number 1 - Default model)

|  | CD | Cognitivereappraisal | PTM |
| --- | --- | --- | --- |
| Cognitivereappraisal | 17.702 | .000 | .000 |
| PTM | 1.133 | .030 | .000 |
| Y1 | .000 | .000 | .928 |
| Y2 | .000 | .000 | 1.084 |
| Y3 | .000 | .000 | 1.000 |
| X1 | 3.628 | .000 | .000 |
| X2 | 2.887 | .000 | .000 |
| X3 | 1.000 | .000 | .000 |

## Standardized Direct Effects (Group number 1 - Default model)

|  | CD | Cognitivereappraisal | PTM |
| --- | --- | --- | --- |
| Cognitivereappraisal | .614 | .000 | .000 |
| PTM | .356 | .271 | .000 |
| Y1 | .000 | .000 | .822 |
| Y2 | .000 | .000 | .895 |
| Y3 | .000 | .000 | .849 |
| X1 | .947 | .000 | .000 |
| X2 | .797 | .000 | .000 |
| X3 | .245 | .000 | .000 |

## Indirect Effects (Group number 1 - Default model)

|  | CD | Cognitivereappraisal | PTM |
| --- | --- | --- | --- |
| Cognitivereappraisal | .000 | .000 | .000 |
| PTM | .529 | .000 | .000 |
| Y1 | 1.542 | .028 | .000 |
| Y2 | 1.802 | .032 | .000 |
| Y3 | 1.662 | .030 | .000 |
| X1 | .000 | .000 | .000 |
| X2 | .000 | .000 | .000 |
| X3 | .000 | .000 | .000 |

## Standardized Indirect Effects (Group number 1 - Default model)

|  | CD | Cognitivereappraisal | PTM |
| --- | --- | --- | --- |
| Cognitivereappraisal | .000 | .000 | .000 |
| PTM | .166 | .000 | .000 |
| Y1 | .430 | .223 | .000 |
| Y2 | .468 | .243 | .000 |
| Y3 | .444 | .230 | .000 |
| X1 | .000 | .000 | .000 |
| X2 | .000 | .000 | .000 |
| X3 | .000 | .000 | .000 |

## Bootstrap (Group number 1 - Default model)

## Bootstrap standard errors (Group number 1 - Default model)

## Scalar Estimates (Group number 1 - Default model)

## Regression Weights: (Group number 1 - Default model)

| Parameter | | | SE | SE-SE | Mean | Bias | SE-Bias |
| --- | --- | --- | --- | --- | --- | --- | --- |
| Cognitivereappraisal | <--- | CD | 7.373 | .369 | 19.332 | 1.630 | .521 |
| PTM | <--- | Cognitivereappraisal | .009 | .000 | .030 | .000 | .001 |
| PTM | <--- | CD | .577 | .029 | 1.260 | .127 | .041 |
| X3 | <--- | CD | .000 | .000 | 1.000 | .000 | .000 |
| X2 | <--- | CD | 1.147 | .057 | 3.161 | .274 | .081 |
| X1 | <--- | CD | 1.488 | .074 | 3.981 | .352 | .105 |
| Y3 | <--- | PTM | .000 | .000 | 1.000 | .000 | .000 |
| Y2 | <--- | PTM | .056 | .003 | 1.093 | .009 | .004 |
| Y1 | <--- | PTM | .067 | .003 | .927 | -.001 | .005 |

## Standardized Regression Weights: (Group number 1 - Default model)

| Parameter | | | SE | SE-SE | Mean | Bias | SE-Bias |
| --- | --- | --- | --- | --- | --- | --- | --- |
| Cognitivereappraisal | <--- | CD | .049 | .002 | .612 | -.001 | .003 |
| PTM | <--- | Cognitivereappraisal | .074 | .004 | .268 | -.003 | .005 |
| PTM | <--- | CD | .068 | .003 | .358 | .001 | .005 |
| X3 | <--- | CD | .062 | .003 | .244 | -.001 | .004 |
| X2 | <--- | CD | .036 | .002 | .797 | .000 | .003 |
| X1 | <--- | CD | .027 | .001 | .947 | .001 | .002 |
| Y3 | <--- | PTM | .023 | .001 | .849 | -.001 | .002 |
| Y2 | <--- | PTM | .018 | .001 | .898 | .003 | .001 |
| Y1 | <--- | PTM | .035 | .002 | .820 | -.002 | .002 |

## Intercepts: (Group number 1 - Default model)

| Parameter | | | SE | SE-SE | Mean | Bias | SE-Bias |
| --- | --- | --- | --- | --- | --- | --- | --- |
| Cognitivereappraisal |  |  | .228 | .011 | 23.621 | -.010 | .016 |
| X3 |  |  | .032 | .002 | 2.526 | .001 | .002 |
| X2 |  |  | .028 | .001 | 3.460 | .002 | .002 |
| X1 |  |  | .031 | .002 | 3.344 | .000 | .002 |
| Y3 |  |  | .212 | .011 | 2.942 | .006 | .015 |
| Y2 |  |  | .218 | .011 | 2.947 | .003 | .015 |
| Y1 |  |  | .211 | .011 | 3.288 | .004 | .015 |

## Variances: (Group number 1 - Default model)

| Parameter | | | SE | SE-SE | Mean | Bias | SE-Bias |
| --- | --- | --- | --- | --- | --- | --- | --- |
| CD |  |  | .017 | .001 | .033 | .002 | .001 |
| E1 |  |  | 1.690 | .084 | 15.937 | -.131 | .119 |
| E2 |  |  | .029 | .001 | .211 | -.002 | .002 |
| E5 |  |  | .034 | .002 | .482 | -.005 | .002 |
| E4 |  |  | .021 | .001 | .147 | -.002 | .001 |
| E3 |  |  | .023 | .001 | .046 | -.001 | .002 |
| E8 |  |  | .014 | .001 | .120 | -.001 | .001 |
| E7 |  |  | .015 | .001 | .089 | -.003 | .001 |
| E6 |  |  | .021 | .001 | .128 | -.001 | .002 |

## Matrices (Group number 1 - Default model)

## Total Effects - Standard Errors (Group number 1 - Default model)

|  | CD | Cognitivereappraisal | PTM |
| --- | --- | --- | --- |
| Cognitivereappraisal | 7.373 | .000 | .000 |
| PTM | .722 | .009 | .000 |
| Y1 | .670 | .008 | .067 |
| Y2 | .775 | .009 | .056 |
| Y3 | .722 | .009 | .000 |
| X1 | 1.488 | .000 | .000 |
| X2 | 1.147 | .000 | .000 |
| X3 | .000 | .000 | .000 |

## Standardized Total Effects - Standard Errors (Group number 1 - Default model)

|  | CD | Cognitivereappraisal | PTM |
| --- | --- | --- | --- |
| Cognitivereappraisal | .049 | .000 | .000 |
| PTM | .050 | .074 | .000 |
| Y1 | .051 | .065 | .035 |
| Y2 | .047 | .067 | .018 |
| Y3 | .046 | .065 | .023 |
| X1 | .027 | .000 | .000 |
| X2 | .036 | .000 | .000 |
| X3 | .062 | .000 | .000 |

## Direct Effects - Standard Errors (Group number 1 - Default model)

|  | CD | Cognitivereappraisal | PTM |
| --- | --- | --- | --- |
| Cognitivereappraisal | 7.373 | .000 | .000 |
| PTM | .577 | .009 | .000 |
| Y1 | .000 | .000 | .067 |
| Y2 | .000 | .000 | .056 |
| Y3 | .000 | .000 | .000 |
| X1 | 1.488 | .000 | .000 |
| X2 | 1.147 | .000 | .000 |
| X3 | .000 | .000 | .000 |

## Standardized Direct Effects - Standard Errors (Group number 1 - Default model)

|  | CD | Cognitivereappraisal | PTM |
| --- | --- | --- | --- |
| Cognitivereappraisal | .049 | .000 | .000 |
| PTM | .068 | .074 | .000 |
| Y1 | .000 | .000 | .035 |
| Y2 | .000 | .000 | .018 |
| Y3 | .000 | .000 | .023 |
| X1 | .027 | .000 | .000 |
| X2 | .036 | .000 | .000 |
| X3 | .062 | .000 | .000 |

## Indirect Effects - Standard Errors (Group number 1 - Default model)

|  | CD | Cognitivereappraisal | PTM |
| --- | --- | --- | --- |
| Cognitivereappraisal | .000 | .000 | .000 |
| PTM | .231 | .000 | .000 |
| Y1 | .670 | .008 | .000 |
| Y2 | .775 | .009 | .000 |
| Y3 | .722 | .009 | .000 |
| X1 | .000 | .000 | .000 |
| X2 | .000 | .000 | .000 |
| X3 | .000 | .000 | .000 |

## Standardized Indirect Effects - Standard Errors (Group number 1 - Default model)

|  | CD | Cognitivereappraisal | PTM |
| --- | --- | --- | --- |
| Cognitivereappraisal | .000 | .000 | .000 |
| PTM | .044 | .000 | .000 |
| Y1 | .051 | .065 | .000 |
| Y2 | .047 | .067 | .000 |
| Y3 | .046 | .065 | .000 |
| X1 | .000 | .000 | .000 |
| X2 | .000 | .000 | .000 |
| X3 | .000 | .000 | .000 |

## Bootstrap Confidence (Group number 1 - Default model)

## Percentile method (Group number 1 - Default model)

## 90% confidence intervals (percentile method)

## Scalar Estimates (Group number 1 - Default model)

## Regression Weights: (Group number 1 - Default model)

| Parameter | | | Estimate | Lower | Upper | P |
| --- | --- | --- | --- | --- | --- | --- |
| Cognitivereappraisal | <--- | CD | 17.702 | 12.436 | 33.086 | .010 |
| PTM | <--- | Cognitivereappraisal | .030 | .015 | .044 | .010 |
| PTM | <--- | CD | 1.133 | .671 | 2.373 | .010 |
| X3 | <--- | CD | 1.000 | 1.000 | 1.000 | ... |
| X2 | <--- | CD | 2.887 | 2.030 | 5.314 | .010 |
| X1 | <--- | CD | 3.628 | 2.471 | 6.583 | .010 |
| Y3 | <--- | PTM | 1.000 | 1.000 | 1.000 | ... |
| Y2 | <--- | PTM | 1.084 | 1.010 | 1.186 | .010 |
| Y1 | <--- | PTM | .928 | .807 | 1.040 | .010 |

## Standardized Regression Weights: (Group number 1 - Default model)

| Parameter | | | Estimate | Lower | Upper | P |
| --- | --- | --- | --- | --- | --- | --- |
| Cognitivereappraisal | <--- | CD | .614 | .526 | .685 | .010 |
| PTM | <--- | Cognitivereappraisal | .271 | .134 | .382 | .010 |
| PTM | <--- | CD | .356 | .251 | .483 | .010 |
| X3 | <--- | CD | .245 | .136 | .349 | .010 |
| X2 | <--- | CD | .797 | .735 | .858 | .010 |
| X1 | <--- | CD | .947 | .900 | .993 | .010 |
| Y3 | <--- | PTM | .849 | .808 | .882 | .010 |
| Y2 | <--- | PTM | .895 | .867 | .928 | .010 |
| Y1 | <--- | PTM | .822 | .761 | .873 | .010 |

## Intercepts: (Group number 1 - Default model)

| Parameter | | | Estimate | Lower | Upper | P |
| --- | --- | --- | --- | --- | --- | --- |
| Cognitivereappraisal |  |  | 23.630 | 23.221 | 23.995 | .010 |
| X3 |  |  | 2.526 | 2.471 | 2.574 | .010 |
| X2 |  |  | 3.458 | 3.406 | 3.507 | .010 |
| X1 |  |  | 3.344 | 3.288 | 3.397 | .010 |
| Y3 |  |  | 2.935 | 2.578 | 3.285 | .010 |
| Y2 |  |  | 2.944 | 2.574 | 3.313 | .010 |
| Y1 |  |  | 3.284 | 2.925 | 3.642 | .010 |

## Variances: (Group number 1 - Default model)

| Parameter | | | Estimate | Lower | Upper | P |
| --- | --- | --- | --- | --- | --- | --- |
| CD |  |  | .031 | .008 | .067 | .010 |
| E1 |  |  | 16.068 | 13.087 | 18.779 | .010 |
| E2 |  |  | .214 | .159 | .264 | .010 |
| E5 |  |  | .487 | .428 | .540 | .010 |
| E4 |  |  | .149 | .111 | .181 | .010 |
| E3 |  |  | .047 | .006 | .086 | .062 |
| E8 |  |  | .121 | .097 | .145 | .010 |
| E7 |  |  | .092 | .062 | .113 | .010 |
| E6 |  |  | .130 | .094 | .170 | .010 |

## Matrices (Group number 1 - Default model)

## Total Effects (Group number 1 - Default model)

## Total Effects - Lower Bounds (PC) (Group number 1 - Default model)

|  | CD | Cognitivereappraisal | PTM |
| --- | --- | --- | --- |
| Cognitivereappraisal | 12.436 | .000 | .000 |
| PTM | 1.066 | .015 | .000 |
| Y1 | .978 | .013 | .807 |
| Y2 | 1.179 | .017 | 1.010 |
| Y3 | 1.066 | .015 | 1.000 |
| X1 | 2.471 | .000 | .000 |
| X2 | 2.030 | .000 | .000 |
| X3 | 1.000 | .000 | .000 |

## Total Effects - Upper Bounds (PC) (Group number 1 - Default model)

|  | CD | Cognitivereappraisal | PTM |
| --- | --- | --- | --- |
| Cognitivereappraisal | 33.086 | .000 | .000 |
| PTM | 3.044 | .044 | .000 |
| Y1 | 2.758 | .043 | 1.040 |
| Y2 | 3.390 | .047 | 1.186 |
| Y3 | 3.044 | .044 | 1.000 |
| X1 | 6.583 | .000 | .000 |
| X2 | 5.314 | .000 | .000 |
| X3 | 1.000 | .000 | .000 |

## Total Effects - Two Tailed Significance (PC) (Group number 1 - Default model)

|  | CD | Cognitivereappraisal | PTM |
| --- | --- | --- | --- |
| Cognitivereappraisal | .010 | ... | ... |
| PTM | .010 | .010 | ... |
| Y1 | .010 | .010 | .010 |
| Y2 | .010 | .010 | .010 |
| Y3 | .010 | .010 | ... |
| X1 | .010 | ... | ... |
| X2 | .010 | ... | ... |
| X3 | ... | ... | ... |

## Standardized Total Effects (Group number 1 - Default model)

## Standardized Total Effects - Lower Bounds (PC) (Group number 1 - Default model)

|  | CD | Cognitivereappraisal | PTM |
| --- | --- | --- | --- |
| Cognitivereappraisal | .526 | .000 | .000 |
| PTM | .438 | .134 | .000 |
| Y1 | .345 | .109 | .761 |
| Y2 | .392 | .123 | .867 |
| Y3 | .371 | .114 | .808 |
| X1 | .900 | .000 | .000 |
| X2 | .735 | .000 | .000 |
| X3 | .136 | .000 | .000 |

## Standardized Total Effects - Upper Bounds (PC) (Group number 1 - Default model)

|  | CD | Cognitivereappraisal | PTM |
| --- | --- | --- | --- |
| Cognitivereappraisal | .685 | .000 | .000 |
| PTM | .600 | .382 | .000 |
| Y1 | .515 | .325 | .873 |
| Y2 | .541 | .344 | .928 |
| Y3 | .512 | .327 | .882 |
| X1 | .993 | .000 | .000 |
| X2 | .858 | .000 | .000 |
| X3 | .349 | .000 | .000 |

## Standardized Total Effects - Two Tailed Significance (PC) (Group number 1 - Default model)

|  | CD | Cognitivereappraisal | PTM |
| --- | --- | --- | --- |
| Cognitivereappraisal | .010 | ... | ... |
| PTM | .010 | .010 | ... |
| Y1 | .010 | .010 | .010 |
| Y2 | .010 | .010 | .010 |
| Y3 | .010 | .010 | .010 |
| X1 | .010 | ... | ... |
| X2 | .010 | ... | ... |
| X3 | .010 | ... | ... |

## Direct Effects (Group number 1 - Default model)

## Direct Effects - Lower Bounds (PC) (Group number 1 - Default model)

|  | CD | Cognitivereappraisal | PTM |
| --- | --- | --- | --- |
| Cognitivereappraisal | 12.436 | .000 | .000 |
| PTM | .671 | .015 | .000 |
| Y1 | .000 | .000 | .807 |
| Y2 | .000 | .000 | 1.010 |
| Y3 | .000 | .000 | 1.000 |
| X1 | 2.471 | .000 | .000 |
| X2 | 2.030 | .000 | .000 |
| X3 | 1.000 | .000 | .000 |

## Direct Effects - Upper Bounds (PC) (Group number 1 - Default model)

|  | CD | Cognitivereappraisal | PTM |
| --- | --- | --- | --- |
| Cognitivereappraisal | 33.086 | .000 | .000 |
| PTM | 2.373 | .044 | .000 |
| Y1 | .000 | .000 | 1.040 |
| Y2 | .000 | .000 | 1.186 |
| Y3 | .000 | .000 | 1.000 |
| X1 | 6.583 | .000 | .000 |
| X2 | 5.314 | .000 | .000 |
| X3 | 1.000 | .000 | .000 |

## Direct Effects - Two Tailed Significance (PC) (Group number 1 - Default model)

|  | CD | Cognitivereappraisal | PTM |
| --- | --- | --- | --- |
| Cognitivereappraisal | .010 | ... | ... |
| PTM | .010 | .010 | ... |
| Y1 | ... | ... | .010 |
| Y2 | ... | ... | .010 |
| Y3 | ... | ... | ... |
| X1 | .010 | ... | ... |
| X2 | .010 | ... | ... |
| X3 | ... | ... | ... |

## Standardized Direct Effects (Group number 1 - Default model)

## Standardized Direct Effects - Lower Bounds (PC) (Group number 1 - Default model)

|  | CD | Cognitivereappraisal | PTM |
| --- | --- | --- | --- |
| Cognitivereappraisal | .526 | .000 | .000 |
| PTM | .251 | .134 | .000 |
| Y1 | .000 | .000 | .761 |
| Y2 | .000 | .000 | .867 |
| Y3 | .000 | .000 | .808 |
| X1 | .900 | .000 | .000 |
| X2 | .735 | .000 | .000 |
| X3 | .136 | .000 | .000 |

## Standardized Direct Effects - Upper Bounds (PC) (Group number 1 - Default model)

|  | CD | Cognitivereappraisal | PTM |
| --- | --- | --- | --- |
| Cognitivereappraisal | .685 | .000 | .000 |
| PTM | .483 | .382 | .000 |
| Y1 | .000 | .000 | .873 |
| Y2 | .000 | .000 | .928 |
| Y3 | .000 | .000 | .882 |
| X1 | .993 | .000 | .000 |
| X2 | .858 | .000 | .000 |
| X3 | .349 | .000 | .000 |

## Standardized Direct Effects - Two Tailed Significance (PC) (Group number 1 - Default model)

|  | CD | Cognitivereappraisal | PTM |
| --- | --- | --- | --- |
| Cognitivereappraisal | .010 | ... | ... |
| PTM | .010 | .010 | ... |
| Y1 | ... | ... | .010 |
| Y2 | ... | ... | .010 |
| Y3 | ... | ... | .010 |
| X1 | .010 | ... | ... |
| X2 | .010 | ... | ... |
| X3 | .010 | ... | ... |

## Indirect Effects (Group number 1 - Default model)

## Indirect Effects - Lower Bounds (PC) (Group number 1 - Default model)

|  | CD | Cognitivereappraisal | PTM |
| --- | --- | --- | --- |
| Cognitivereappraisal | .000 | .000 | .000 |
| PTM | .283 | .000 | .000 |
| Y1 | .978 | .013 | .000 |
| Y2 | 1.179 | .017 | .000 |
| Y3 | 1.066 | .015 | .000 |
| X1 | .000 | .000 | .000 |
| X2 | .000 | .000 | .000 |
| X3 | .000 | .000 | .000 |

## Indirect Effects - Upper Bounds (PC) (Group number 1 - Default model)

|  | CD | Cognitivereappraisal | PTM |
| --- | --- | --- | --- |
| Cognitivereappraisal | .000 | .000 | .000 |
| PTM | .933 | .000 | .000 |
| Y1 | 2.758 | .043 | .000 |
| Y2 | 3.390 | .047 | .000 |
| Y3 | 3.044 | .044 | .000 |
| X1 | .000 | .000 | .000 |
| X2 | .000 | .000 | .000 |
| X3 | .000 | .000 | .000 |

## Indirect Effects - Two Tailed Significance (PC) (Group number 1 - Default model)

|  | CD | Cognitivereappraisal | PTM |
| --- | --- | --- | --- |
| Cognitivereappraisal | ... | ... | ... |
| PTM | .010 | ... | ... |
| Y1 | .010 | .010 | ... |
| Y2 | .010 | .010 | ... |
| Y3 | .010 | .010 | ... |
| X1 | ... | ... | ... |
| X2 | ... | ... | ... |
| X3 | ... | ... | ... |

## Standardized Indirect Effects (Group number 1 - Default model)

## Standardized Indirect Effects - Lower Bounds (PC) (Group number 1 - Default model)

|  | CD | Cognitivereappraisal | PTM |
| --- | --- | --- | --- |
| Cognitivereappraisal | .000 | .000 | .000 |
| PTM | .086 | .000 | .000 |
| Y1 | .345 | .109 | .000 |
| Y2 | .392 | .123 | .000 |
| Y3 | .371 | .114 | .000 |
| X1 | .000 | .000 | .000 |
| X2 | .000 | .000 | .000 |
| X3 | .000 | .000 | .000 |

## Standardized Indirect Effects - Upper Bounds (PC) (Group number 1 - Default model)

|  | CD | Cognitivereappraisal | PTM |
| --- | --- | --- | --- |
| Cognitivereappraisal | .000 | .000 | .000 |
| PTM | .234 | .000 | .000 |
| Y1 | .515 | .325 | .000 |
| Y2 | .541 | .344 | .000 |
| Y3 | .512 | .327 | .000 |
| X1 | .000 | .000 | .000 |
| X2 | .000 | .000 | .000 |
| X3 | .000 | .000 | .000 |

## Standardized Indirect Effects - Two Tailed Significance (PC) (Group number 1 - Default model)

|  | CD | Cognitivereappraisal | PTM |
| --- | --- | --- | --- |
| Cognitivereappraisal | ... | ... | ... |
| PTM | .010 | ... | ... |
| Y1 | .010 | .010 | ... |
| Y2 | .010 | .010 | ... |
| Y3 | .010 | .010 | ... |
| X1 | ... | ... | ... |
| X2 | ... | ... | ... |
| X3 | ... | ... | ... |

## Bias-corrected percentile method (Group number 1 - Default model)

## 90% confidence intervals (bias-corrected percentile method)

## Scalar Estimates (Group number 1 - Default model)

## Regression Weights: (Group number 1 - Default model)

| Parameter | | | Estimate | Lower | Upper | P |
| --- | --- | --- | --- | --- | --- | --- |
| Cognitivereappraisal | <--- | CD | 17.702 | 12.703 | 33.393 | .007 |
| PTM | <--- | Cognitivereappraisal | .030 | .016 | .045 | .007 |
| PTM | <--- | CD | 1.133 | .679 | 2.409 | .009 |
| X3 | <--- | CD | 1.000 | 1.000 | 1.000 | ... |
| X2 | <--- | CD | 2.887 | 2.015 | 5.194 | .012 |
| X1 | <--- | CD | 3.628 | 2.599 | 7.426 | .007 |
| Y3 | <--- | PTM | 1.000 | 1.000 | 1.000 | ... |
| Y2 | <--- | PTM | 1.084 | 1.002 | 1.176 | .014 |
| Y1 | <--- | PTM | .928 | .794 | 1.021 | .019 |

## Standardized Regression Weights: (Group number 1 - Default model)

| Parameter | | | Estimate | Lower | Upper | P |
| --- | --- | --- | --- | --- | --- | --- |
| Cognitivereappraisal | <--- | CD | .614 | .519 | .680 | .016 |
| PTM | <--- | Cognitivereappraisal | .271 | .133 | .380 | .011 |
| PTM | <--- | CD | .356 | .255 | .493 | .008 |
| X3 | <--- | CD | .245 | .131 | .339 | .013 |
| X2 | <--- | CD | .797 | .738 | .859 | .007 |
| X1 | <--- | CD | .947 | .902 | .995 | .008 |
| Y3 | <--- | PTM | .849 | .807 | .882 | .011 |
| Y2 | <--- | PTM | .895 | .860 | .920 | .030 |
| Y1 | <--- | PTM | .822 | .757 | .871 | .012 |

## Intercepts: (Group number 1 - Default model)

| Parameter | | | Estimate | Lower | Upper | P |
| --- | --- | --- | --- | --- | --- | --- |
| Cognitivereappraisal |  |  | 23.630 | 23.229 | 24.015 | .007 |
| X3 |  |  | 2.526 | 2.467 | 2.569 | .015 |
| X2 |  |  | 3.458 | 3.402 | 3.498 | .020 |
| X1 |  |  | 3.344 | 3.289 | 3.404 | .008 |
| Y3 |  |  | 2.935 | 2.553 | 3.264 | .015 |
| Y2 |  |  | 2.944 | 2.583 | 3.335 | .008 |
| Y1 |  |  | 3.284 | 2.907 | 3.619 | .014 |

## Variances: (Group number 1 - Default model)

| Parameter | | | Estimate | Lower | Upper | P |
| --- | --- | --- | --- | --- | --- | --- |
| CD |  |  | .031 | .008 | .067 | .010 |
| E1 |  |  | 16.068 | 13.697 | 19.293 | .003 |
| E2 |  |  | .214 | .168 | .272 | .005 |
| E5 |  |  | .487 | .440 | .556 | .004 |
| E4 |  |  | .149 | .115 | .186 | .005 |
| E3 |  |  | .047 | .006 | .087 | .059 |
| E8 |  |  | .121 | .099 | .146 | .005 |
| E7 |  |  | .092 | .076 | .130 | .001 |
| E6 |  |  | .130 | .099 | .174 | .005 |

## Matrices (Group number 1 - Default model)

## Total Effects (Group number 1 - Default model)

## Total Effects - Lower Bounds (BC) (Group number 1 - Default model)

|  | CD | Cognitivereappraisal | PTM |
| --- | --- | --- | --- |
| Cognitivereappraisal | 12.703 | .000 | .000 |
| PTM | 1.187 | .016 | .000 |
| Y1 | .975 | .014 | .794 |
| Y2 | 1.218 | .017 | 1.002 |
| Y3 | 1.187 | .016 | 1.000 |
| X1 | 2.599 | .000 | .000 |
| X2 | 2.015 | .000 | .000 |
| X3 | 1.000 | .000 | .000 |

## Total Effects - Upper Bounds (BC) (Group number 1 - Default model)

|  | CD | Cognitivereappraisal | PTM |
| --- | --- | --- | --- |
| Cognitivereappraisal | 33.393 | .000 | .000 |
| PTM | 3.324 | .045 | .000 |
| Y1 | 2.745 | .043 | 1.021 |
| Y2 | 3.554 | .047 | 1.176 |
| Y3 | 3.324 | .045 | 1.000 |
| X1 | 7.426 | .000 | .000 |
| X2 | 5.194 | .000 | .000 |
| X3 | 1.000 | .000 | .000 |

## Total Effects - Two Tailed Significance (BC) (Group number 1 - Default model)

|  | CD | Cognitivereappraisal | PTM |
| --- | --- | --- | --- |
| Cognitivereappraisal | .007 | ... | ... |
| PTM | .005 | .007 | ... |
| Y1 | .011 | .009 | .019 |
| Y2 | .007 | .012 | .014 |
| Y3 | .005 | .007 | ... |
| X1 | .007 | ... | ... |
| X2 | .012 | ... | ... |
| X3 | ... | ... | ... |

## Standardized Total Effects (Group number 1 - Default model)

## Standardized Total Effects - Lower Bounds (BC) (Group number 1 - Default model)

|  | CD | Cognitivereappraisal | PTM |
| --- | --- | --- | --- |
| Cognitivereappraisal | .519 | .000 | .000 |
| PTM | .436 | .133 | .000 |
| Y1 | .338 | .111 | .757 |
| Y2 | .389 | .125 | .860 |
| Y3 | .371 | .114 | .807 |
| X1 | .902 | .000 | .000 |
| X2 | .738 | .000 | .000 |
| X3 | .131 | .000 | .000 |

## Standardized Total Effects - Upper Bounds (BC) (Group number 1 - Default model)

|  | CD | Cognitivereappraisal | PTM |
| --- | --- | --- | --- |
| Cognitivereappraisal | .680 | .000 | .000 |
| PTM | .598 | .380 | .000 |
| Y1 | .504 | .327 | .871 |
| Y2 | .536 | .351 | .920 |
| Y3 | .512 | .327 | .882 |
| X1 | .995 | .000 | .000 |
| X2 | .859 | .000 | .000 |
| X3 | .339 | .000 | .000 |

## Standardized Total Effects - Two Tailed Significance (BC) (Group number 1 - Default model)

|  | CD | Cognitivereappraisal | PTM |
| --- | --- | --- | --- |
| Cognitivereappraisal | .016 | ... | ... |
| PTM | .012 | .011 | ... |
| Y1 | .014 | .008 | .012 |
| Y2 | .014 | .009 | .030 |
| Y3 | .010 | .010 | .011 |
| X1 | .008 | ... | ... |
| X2 | .007 | ... | ... |
| X3 | .013 | ... | ... |

## Direct Effects (Group number 1 - Default model)

## Direct Effects - Lower Bounds (BC) (Group number 1 - Default model)

|  | CD | Cognitivereappraisal | PTM |
| --- | --- | --- | --- |
| Cognitivereappraisal | 12.703 | .000 | .000 |
| PTM | .679 | .016 | .000 |
| Y1 | .000 | .000 | .794 |
| Y2 | .000 | .000 | 1.002 |
| Y3 | .000 | .000 | 1.000 |
| X1 | 2.599 | .000 | .000 |
| X2 | 2.015 | .000 | .000 |
| X3 | 1.000 | .000 | .000 |

## Direct Effects - Upper Bounds (BC) (Group number 1 - Default model)

|  | CD | Cognitivereappraisal | PTM |
| --- | --- | --- | --- |
| Cognitivereappraisal | 33.393 | .000 | .000 |
| PTM | 2.409 | .045 | .000 |
| Y1 | .000 | .000 | 1.021 |
| Y2 | .000 | .000 | 1.176 |
| Y3 | .000 | .000 | 1.000 |
| X1 | 7.426 | .000 | .000 |
| X2 | 5.194 | .000 | .000 |
| X3 | 1.000 | .000 | .000 |

## Direct Effects - Two Tailed Significance (BC) (Group number 1 - Default model)

|  | CD | Cognitivereappraisal | PTM |
| --- | --- | --- | --- |
| Cognitivereappraisal | .007 | ... | ... |
| PTM | .009 | .007 | ... |
| Y1 | ... | ... | .019 |
| Y2 | ... | ... | .014 |
| Y3 | ... | ... | ... |
| X1 | .007 | ... | ... |
| X2 | .012 | ... | ... |
| X3 | ... | ... | ... |

## Standardized Direct Effects (Group number 1 - Default model)

## Standardized Direct Effects - Lower Bounds (BC) (Group number 1 - Default model)

|  | CD | Cognitivereappraisal | PTM |
| --- | --- | --- | --- |
| Cognitivereappraisal | .519 | .000 | .000 |
| PTM | .255 | .133 | .000 |
| Y1 | .000 | .000 | .757 |
| Y2 | .000 | .000 | .860 |
| Y3 | .000 | .000 | .807 |
| X1 | .902 | .000 | .000 |
| X2 | .738 | .000 | .000 |
| X3 | .131 | .000 | .000 |

## Standardized Direct Effects - Upper Bounds (BC) (Group number 1 - Default model)

|  | CD | Cognitivereappraisal | PTM |
| --- | --- | --- | --- |
| Cognitivereappraisal | .680 | .000 | .000 |
| PTM | .493 | .380 | .000 |
| Y1 | .000 | .000 | .871 |
| Y2 | .000 | .000 | .920 |
| Y3 | .000 | .000 | .882 |
| X1 | .995 | .000 | .000 |
| X2 | .859 | .000 | .000 |
| X3 | .339 | .000 | .000 |

## Standardized Direct Effects - Two Tailed Significance (BC) (Group number 1 - Default model)

|  | CD | Cognitivereappraisal | PTM |
| --- | --- | --- | --- |
| Cognitivereappraisal | .016 | ... | ... |
| PTM | .008 | .011 | ... |
| Y1 | ... | ... | .012 |
| Y2 | ... | ... | .030 |
| Y3 | ... | ... | .011 |
| X1 | .008 | ... | ... |
| X2 | .007 | ... | ... |
| X3 | .013 | ... | ... |

## Indirect Effects (Group number 1 - Default model)

## Indirect Effects - Lower Bounds (BC) (Group number 1 - Default model)

|  | CD | Cognitivereappraisal | PTM |
| --- | --- | --- | --- |
| Cognitivereappraisal | .000 | .000 | .000 |
| PTM | .283 | .000 | .000 |
| Y1 | .975 | .014 | .000 |
| Y2 | 1.218 | .017 | .000 |
| Y3 | 1.187 | .016 | .000 |
| X1 | .000 | .000 | .000 |
| X2 | .000 | .000 | .000 |
| X3 | .000 | .000 | .000 |

## Indirect Effects - Upper Bounds (BC) (Group number 1 - Default model)

|  | CD | Cognitivereappraisal | PTM |
| --- | --- | --- | --- |
| Cognitivereappraisal | .000 | .000 | .000 |
| PTM | .933 | .000 | .000 |
| Y1 | 2.745 | .043 | .000 |
| Y2 | 3.554 | .047 | .000 |
| Y3 | 3.324 | .045 | .000 |
| X1 | .000 | .000 | .000 |
| X2 | .000 | .000 | .000 |
| X3 | .000 | .000 | .000 |

## Indirect Effects - Two Tailed Significance (BC) (Group number 1 - Default model)

|  | CD | Cognitivereappraisal | PTM |
| --- | --- | --- | --- |
| Cognitivereappraisal | ... | ... | ... |
| PTM | .010 | ... | ... |
| Y1 | .011 | .009 | ... |
| Y2 | .007 | .012 | ... |
| Y3 | .005 | .007 | ... |
| X1 | ... | ... | ... |
| X2 | ... | ... | ... |
| X3 | ... | ... | ... |

## Standardized Indirect Effects (Group number 1 - Default model)

## Standardized Indirect Effects - Lower Bounds (BC) (Group number 1 - Default model)

|  | CD | Cognitivereappraisal | PTM |
| --- | --- | --- | --- |
| Cognitivereappraisal | .000 | .000 | .000 |
| PTM | .088 | .000 | .000 |
| Y1 | .338 | .111 | .000 |
| Y2 | .389 | .125 | .000 |
| Y3 | .371 | .114 | .000 |
| X1 | .000 | .000 | .000 |
| X2 | .000 | .000 | .000 |
| X3 | .000 | .000 | .000 |

## Standardized Indirect Effects - Upper Bounds (BC) (Group number 1 - Default model)

|  | CD | Cognitivereappraisal | PTM |
| --- | --- | --- | --- |
| Cognitivereappraisal | .000 | .000 | .000 |
| PTM | .237 | .000 | .000 |
| Y1 | .504 | .327 | .000 |
| Y2 | .536 | .351 | .000 |
| Y3 | .512 | .327 | .000 |
| X1 | .000 | .000 | .000 |
| X2 | .000 | .000 | .000 |
| X3 | .000 | .000 | .000 |

## Standardized Indirect Effects - Two Tailed Significance (BC) (Group number 1 - Default model)

|  | CD | Cognitivereappraisal | PTM |
| --- | --- | --- | --- |
| Cognitivereappraisal | ... | ... | ... |
| PTM | .009 | ... | ... |
| Y1 | .014 | .008 | ... |
| Y2 | .014 | .009 | ... |
| Y3 | .010 | .010 | ... |
| X1 | ... | ... | ... |
| X2 | ... | ... | ... |
| X3 | ... | ... | ... |

## Minimization History (Default model)

| Iteration |  | Negative eigenvalues | Condition # | Smallest eigenvalue | Diameter | F | NTries | Ratio |
| --- | --- | --- | --- | --- | --- | --- | --- | --- |
| 0 | e | 4 |  | -.392 | 9999.000 | 1501.687 | 0 | 9999.000 |
| 1 | e | 4 |  | -1.328 | 1.180 | 992.184 | 19 | .619 |
| 2 | e | 2 |  | -.322 | .520 | 635.185 | 5 | .779 |
| 3 | e | 2 |  | -.191 | .357 | 471.222 | 6 | .757 |
| 4 | e\* | 0 | 4982.752 |  | 1.141 | 171.897 | 7 | .762 |
| 5 | e | 1 |  | -.510 | .670 | 147.116 | 3 | .000 |
| 6 | e | 0 | 1158.118 |  | .248 | 102.612 | 5 | .758 |
| 7 | e | 0 | 1114.183 |  | .868 | 101.568 | 1 | .022 |
| 8 | e | 0 | 2865.067 |  | .531 | 38.052 | 1 | 1.120 |
| 9 | e | 0 | 7295.426 |  | .511 | 28.034 | 1 | 1.231 |
| 10 | e | 0 | 9142.370 |  | .625 | 24.489 | 1 | 1.091 |
| 11 | e | 0 | 25368.745 |  | .388 | 22.361 | 1 | 1.212 |
| 12 | e | 0 | 23109.780 |  | .538 | 21.741 | 1 | .918 |
| 13 | e | 0 | 58530.539 |  | .206 | 21.374 | 1 | 1.103 |
| 14 | e | 0 | 64990.593 |  | .221 | 21.325 | 1 | 1.029 |
| 15 | e | 0 | 80676.684 |  | .036 | 21.320 | 1 | 1.026 |
| 16 | e | 0 | 80747.794 |  | .006 | 21.320 | 1 | 1.004 |
| 17 | e | 0 | 80995.800 |  | .000 | 21.320 | 1 | 1.000 |

## Bootstrap (Default model)

## Summary of Bootstrap Iterations (Default model)

## (Default model)

| Iterations | Method 0 | Method 1 | Method 2 |
| --- | --- | --- | --- |
| 1 | 0 | 0 | 0 |
| 2 | 0 | 0 | 0 |
| 3 | 0 | 0 | 0 |
| 4 | 0 | 0 | 0 |
| 5 | 0 | 0 | 0 |
| 6 | 0 | 0 | 0 |
| 7 | 0 | 0 | 0 |
| 8 | 0 | 2 | 0 |
| 9 | 0 | 2 | 0 |
| 10 | 0 | 9 | 0 |
| 11 | 0 | 12 | 0 |
| 12 | 0 | 18 | 0 |
| 13 | 0 | 25 | 0 |
| 14 | 0 | 15 | 0 |
| 15 | 0 | 18 | 0 |
| 16 | 0 | 17 | 0 |
| 17 | 0 | 13 | 0 |
| 18 | 0 | 10 | 0 |
| 19 | 0 | 58 | 1 |
| Total | 0 | 199 | 1 |

0 bootstrap samples were unused because of a singular covariance matrix.

0 bootstrap samples were unused because a solution was not found.

200 usable bootstrap samples were obtained.

## Bootstrap Distributions (Default model)

## ML discrepancy (implied vs sample) (Default model)

|  |  |  |
| --- | --- | --- |
|  |  | \|-------------------- |
|  | 8.315 | \|\* |
|  | 13.598 | \|\*\*\* |
|  | 18.881 | \|\*\*\*\*\*\*\*\* |
|  | 24.163 | \|\*\*\*\*\*\*\*\*\*\*\*\*\*\*\* |
|  | 29.446 | \|\*\*\*\*\*\*\*\*\*\*\*\*\*\*\*\*\*\* |
|  | 34.729 | \|\*\*\*\*\*\*\*\*\*\*\*\*\*\* |
|  | 40.012 | \|\*\*\*\*\*\*\*\*\*\*\*\*\*\*\*\* |
| N = 200 | 45.294 | \|\*\*\*\*\*\*\* |
| Mean = 35.565 | 50.577 | \|\*\*\*\*\*\*\*\*\*\*\* |
| S. e. = .948 | 55.860 | \|\*\* |
|  | 61.142 | \|\*\*\* |
|  | 66.425 | \|\* |
|  | 71.708 | \|\* |
|  | 76.991 | \|\* |
|  | 82.273 | \|\* |
|  |  | \|-------------------- |

## ML discrepancy (implied vs pop) (Default model)

|  |  |  |
| --- | --- | --- |
|  |  | \|-------------------- |
|  | 31.310 | \|\* |
|  | 36.310 | \|\*\*\*\*\*\* |
|  | 41.311 | \|\*\*\*\*\*\*\*\*\*\*\*\* |
|  | 46.311 | \|\*\*\*\*\*\*\*\*\*\*\*\*\* |
|  | 51.311 | \|\*\*\*\*\*\*\*\*\*\*\*\*\*\*\*\*\*\*\*\* |
|  | 56.311 | \|\*\*\*\*\*\*\*\*\*\*\*\*\*\*\* |
|  | 61.311 | \|\*\*\*\*\*\*\*\*\*\*\* |
| N = 200 | 66.311 | \|\*\*\*\*\*\*\* |
| Mean = 56.303 | 71.311 | \|\*\*\*\*\* |
| S. e. = .941 | 76.311 | \|\*\*\*\*\* |
|  | 81.311 | \|\*\*\*\* |
|  | 86.312 | \|\* |
|  | 91.312 | \|\* |
|  | 96.312 | \|\* |
|  | 101.312 | \|\* |
|  |  | \|-------------------- |

## K-L overoptimism (unstabilized) (Default model)

|  |  |  |
| --- | --- | --- |
|  |  | \|-------------------- |
|  | -232.699 | \|\* |
|  | -183.830 | \|\*\*\* |
|  | -134.960 | \|\*\*\*\* |
|  | -86.090 | \|\*\*\*\*\*\*\*\*\* |
|  | -37.221 | \|\*\*\*\*\*\*\*\*\*\*\*\* |
|  | 11.649 | \|\*\*\*\*\*\*\*\*\*\*\*\*\*\* |
|  | 60.519 | \|\*\*\*\*\*\*\*\*\*\*\*\*\* |
| N = 200 | 109.388 | \|\*\*\*\*\*\*\*\*\*\*\*\* |
| Mean = 80.030 | 158.258 | \|\*\*\*\*\*\*\*\*\*\*\* |
| S. e. = 9.857 | 207.128 | \|\*\*\*\*\*\*\* |
|  | 255.997 | \|\*\*\*\*\*\*\*\*\* |
|  | 304.867 | \|\*\*\* |
|  | 353.737 | \|\*\*\* |
|  | 402.607 | \|\* |
|  | 451.476 | \|\*\* |
|  |  | \|-------------------- |

## K-L overoptimism (stabilized) (Default model)

|  |  |  |
| --- | --- | --- |
|  |  | \|-------------------- |
|  | 18.036 | \|\* |
|  | 27.876 | \|\*\*\*\*\*\* |
|  | 37.716 | \|\*\*\*\*\*\*\* |
|  | 47.556 | \|\*\*\*\*\*\*\*\*\*\*\*\*\*\*\* |
|  | 57.396 | \|\*\*\*\*\*\*\*\*\*\*\*\*\*\*\*\* |
|  | 67.236 | \|\*\*\*\*\*\*\*\*\*\*\*\*\*\*\*\* |
|  | 77.076 | \|\*\*\*\*\*\*\*\*\*\*\*\*\*\*\*\* |
| N = 200 | 86.915 | \|\*\*\*\*\*\* |
| Mean = 69.417 | 96.755 | \|\*\*\*\*\* |
| S. e. = 1.899 | 106.595 | \|\*\*\*\*\*\* |
|  | 116.435 | \|\*\*\* |
|  | 126.275 | \|\*\*\*\* |
|  | 136.115 | \|\*\* |
|  | 145.955 | \| |
|  | 155.795 | \|\* |
|  |  | \|-------------------- |

## Model Fit Summary

## CMIN

| Model | NPAR | CMIN | DF | P | CMIN/DF |
| --- | --- | --- | --- | --- | --- |
| Default model | 23 | 21.320 | 12 | .046 | 1.777 |
| Saturated model | 35 | .000 | 0 |  |  |
| Independence model | 14 | 1410.665 | 21 | .000 | 67.175 |

## Baseline Comparisons

| Model | NFI Delta1 | RFI rho1 | IFI Delta2 | TLI rho2 | CFI |
| --- | --- | --- | --- | --- | --- |
| Default model | .985 | .974 | .993 | .988 | .993 |
| Saturated model | 1.000 |  | 1.000 |  | 1.000 |
| Independence model | .000 | .000 | .000 | .000 | .000 |

## Parsimony-Adjusted Measures

| Model | PRATIO | PNFI | PCFI |
| --- | --- | --- | --- |
| Default model | .571 | .563 | .568 |
| Saturated model | .000 | .000 | .000 |
| Independence model | 1.000 | .000 | .000 |

## NCP

| Model | NCP | LO 90 | HI 90 |
| --- | --- | --- | --- |
| Default model | 9.320 | .168 | 26.272 |
| Saturated model | .000 | .000 | .000 |
| Independence model | 1389.665 | 1270.213 | 1516.490 |

## FMIN

| Model | FMIN | F0 | LO 90 | HI 90 |
| --- | --- | --- | --- | --- |
| Default model | .052 | .023 | .000 | .064 |
| Saturated model | .000 | .000 | .000 | .000 |
| Independence model | 3.441 | 3.389 | 3.098 | 3.699 |

## RMSEA

| Model | RMSEA | LO 90 | HI 90 | PCLOSE |
| --- | --- | --- | --- | --- |
| Default model | .044 | .006 | .073 | .600 |
| Independence model | .402 | .384 | .420 | .000 |

## AIC

| Model | AIC | BCC | BIC | CAIC |
| --- | --- | --- | --- | --- |
| Default model | 67.320 | 68.236 |  |  |
| Saturated model | 70.000 | 71.393 |  |  |
| Independence model | 1438.665 | 1439.222 |  |  |

## ECVI

| Model | ECVI | LO 90 | HI 90 | MECVI |
| --- | --- | --- | --- | --- |
| Default model | .164 | .142 | .206 | .166 |
| Saturated model | .171 | .171 | .171 | .174 |
| Independence model | 3.509 | 3.218 | 3.818 | 3.510 |

## HOELTER

| Model | HOELTER .05 | HOELTER .01 |
| --- | --- | --- |
| Default model | 405 | 505 |
| Independence model | 10 | 12 |

## Execution time summary

|  |  |
| --- | --- |
| Minimization: | .020 |
| Miscellaneous: | .121 |
| Bootstrap: | .085 |
| Total: | .226 |
