# Supplementary material for: The indirect association between psychological resilience and prosocial behavior through cognitive reappraisal in Chinese college students
Source: Front Psychol. 2026 Sep 7;17:1789130. doi: 10.3389/fpsyg.2026.1789130 (PMC13591386; doi:10.3389/fpsyg.2026.1789130)
Supplement: Supplementary file 1 [file Data_Sheet_1.zip › 陈静数据分析/aoms数据/model fit/Model fit.docx]

Model fit

|  | *χ2/df* | NFI | RFI | IFI | TLI | CFI | RMSEA |
| --- | --- | --- | --- | --- | --- | --- | --- |
| X→M→Y | 1.777 | 0.985 | 0.974 | 0.993 | 0.988 | 0.993 | 0.044 |
