# Supplementary material for: The indirect association between psychological resilience and prosocial behavior through cognitive reappraisal in Chinese college students
Source: Front Psychol. 2026 Sep 7;17:1789130. doi: 10.3389/fpsyg.2026.1789130 (PMC13591386; doi:10.3389/fpsyg.2026.1789130)
Supplement: Supplementary file 1 [file Data_Sheet_1.zip › 陈静数据分析/aoms数据/pppp/pppp.AmosOutput]

pppp.amw


# C:\Users\86189\Desktop\pppp.amw

## Analysis Summary

## Date and Time

Date: 2025年7月24日

Time: 0:42:18

## Title

pppp: 2025年7月24日 0:42

## Groups

## Group number 1 (Group number 1)

## Notes for Group (Group number 1)

The model is recursive.

Sample size = 411

## Variable Summary (Group number 1)

## Your model contains the following variables (Group number 1)

Observed, endogenous variables

c17

c24

c23

c16

c18

c12

c22

c11

c14

c21

c9

c8

c7

c6

c5

c4

c2

c1

c3

c20

c25

c10

c15

c19

c13

Unobserved, exogenous variables

F1

e1

e2

e3

e4

e5

e6

e7

e8

e9

e10

F2

e12

e13

e14

e15

e16

e17

e18

e19

F3

e20

e21

e22

e23

e24

e25

e26

## Variable counts (Group number 1)

|  |  |
| --- | --- |
| Number of variables in your model: | 53 |
| Number of observed variables: | 25 |
| Number of unobserved variables: | 28 |
| Number of exogenous variables: | 28 |
| Number of endogenous variables: | 25 |

## Parameter Summary (Group number 1)

|  | Weights | Covariances | Variances | Means | Intercepts | Total |
| --- | --- | --- | --- | --- | --- | --- |
| Fixed | 27 | 0 | 0 | 0 | 0 | 27 |
| Labeled | 0 | 0 | 0 | 0 | 0 | 0 |
| Unlabeled | 23 | 3 | 28 | 0 | 0 | 54 |
| Total | 50 | 3 | 28 | 0 | 0 | 81 |

## Models

## Default model (Default model)

## Notes for Model (Default model)

## Computation of degrees of freedom (Default model)

|  |  |
| --- | --- |
| Number of distinct sample moments: | 325 |
| Number of distinct parameters to be estimated: | 54 |
| Degrees of freedom (325 - 54): | 271 |

## Result (Default model)

The model is probably unidentified. In order to achieve identifiability, it will probably be necessary to impose 1 additional constraint.

## Group number 1 (Group number 1 - Default model)

## Estimates (Group number 1 - Default model)

## Scalar Estimates (Group number 1 - Default model)

The (probably) unidentified parameters are marked.

## Regression Weights: (Group number 1 - Default model)

|  |  |  |  |
| --- | --- | --- | --- |
| c17 | <--- | F1 |  |
| c24 | <--- | F1 |  |
| c23 | <--- | F1 |  |
| c16 | <--- | F1 |  |
| c18 | <--- | F1 |  |
| c12 | <--- | F1 |  |
| c22 | <--- | F1 |  |
| c11 | <--- | F1 |  |
| c14 | <--- | F1 |  |
| c21 | <--- | F1 |  |
| c9 | <--- | F2 | unidentified |
| c8 | <--- | F2 | unidentified |
| c7 | <--- | F2 | unidentified |
| c6 | <--- | F2 | unidentified |
| c5 | <--- | F2 | unidentified |
| c4 | <--- | F2 | unidentified |
| c2 | <--- | F2 | unidentified |
| c1 | <--- | F2 | unidentified |
| c3 | <--- | F3 |  |
| c20 | <--- | F3 |  |
| c25 | <--- | F1 |  |
| c10 | <--- | F1 |  |
| c15 | <--- | F1 |  |
| c19 | <--- | F1 |  |
| c13 | <--- | F1 |  |

## Covariances: (Group number 1 - Default model)

|  |  |  |  |
| --- | --- | --- | --- |
| F1 | <--> | F2 | unidentified |
| F1 | <--> | F3 |  |
| F2 | <--> | F3 | unidentified |

## Variances: (Group number 1 - Default model)

|  |  |  |  |
| --- | --- | --- | --- |
| F1 |  |  |  |
| F2 |  |  | unidentified |
| F3 |  |  |  |
| e1 |  |  |  |
| e2 |  |  |  |
| e3 |  |  |  |
| e4 |  |  |  |
| e5 |  |  |  |
| e6 |  |  |  |
| e7 |  |  |  |
| e8 |  |  |  |
| e9 |  |  |  |
| e10 |  |  |  |
| e12 |  |  |  |
| e13 |  |  |  |
| e14 |  |  |  |
| e15 |  |  |  |
| e16 |  |  |  |
| e17 |  |  |  |
| e18 |  |  |  |
| e19 |  |  |  |
| e20 |  |  |  |
| e21 |  |  |  |
| e22 |  |  |  |
| e23 |  |  |  |
| e24 |  |  |  |
| e25 |  |  |  |
| e26 |  |  |  |

## Minimization History (Default model)

| Iteration |  | Negative eigenvalues | Condition # | Smallest eigenvalue | Diameter | F | NTries | Ratio |
| --- | --- | --- | --- | --- | --- | --- | --- | --- |
| 0 | e | 6 |  | -2.102 | 9999.000 | 4507.826 | 0 | 9999.000 |
| 1 | e\* | 4 |  | -.141 | 3.622 | 2020.335 | 20 | .166 |

## Model Fit Summary

## CMIN

| Model | NPAR | CMIN | DF | P | CMIN/DF |
| --- | --- | --- | --- | --- | --- |
| Saturated model | 325 | .000 | 0 |  |  |
| Independence model | 25 | 4848.808 | 300 | .000 | 16.163 |

## RMR, GFI

| Model | RMR | GFI | AGFI | PGFI |
| --- | --- | --- | --- | --- |
| Saturated model | .000 | 1.000 |  |  |
| Independence model | .335 | .219 | .154 | .202 |

## Baseline Comparisons

| Model | NFI Delta1 | RFI rho1 | IFI Delta2 | TLI rho2 | CFI |
| --- | --- | --- | --- | --- | --- |
| Saturated model | 1.000 |  | 1.000 |  | 1.000 |
| Independence model | .000 | .000 | .000 | .000 | .000 |

## Parsimony-Adjusted Measures

| Model | PRATIO | PNFI | PCFI |
| --- | --- | --- | --- |
| Saturated model | .000 | .000 | .000 |
| Independence model | 1.000 | .000 | .000 |

## NCP

| Model | NCP | LO 90 | HI 90 |
| --- | --- | --- | --- |
| Saturated model | .000 | .000 | .000 |
| Independence model | 4548.808 | 4326.851 | 4778.034 |

## FMIN

| Model | FMIN | F0 | LO 90 | HI 90 |
| --- | --- | --- | --- | --- |
| Saturated model | .000 | .000 | .000 | .000 |
| Independence model | 11.826 | 11.095 | 10.553 | 11.654 |

## RMSEA

| Model | RMSEA | LO 90 | HI 90 | PCLOSE |
| --- | --- | --- | --- | --- |
| Independence model | .192 | .188 | .197 | .000 |

## AIC

| Model | AIC | BCC | BIC | CAIC |
| --- | --- | --- | --- | --- |
| Saturated model | 650.000 | 694.010 | 1956.043 | 2281.043 |
| Independence model | 4898.808 | 4902.194 | 4999.273 | 5024.273 |

## ECVI

| Model | ECVI | LO 90 | HI 90 | MECVI |
| --- | --- | --- | --- | --- |
| Saturated model | 1.585 | 1.585 | 1.585 | 1.693 |
| Independence model | 11.948 | 11.407 | 12.507 | 11.957 |

## HOELTER

| Model | HOELTER .05 | HOELTER .01 |
| --- | --- | --- |
| Independence model | 29 | 31 |

## Execution time summary

|  |  |
| --- | --- |
| Minimization: | .002 |
| Miscellaneous: | .355 |
| Bootstrap: | .000 |
| Total: | .357 |
