# Supplementary material for: The indirect association between psychological resilience and prosocial behavior through cognitive reappraisal in Chinese college students
Source: Front Psychol. 2026 Sep 7;17:1789130. doi: 10.3389/fpsyg.2026.1789130 (PMC13591386; doi:10.3389/fpsyg.2026.1789130)
Supplement: Supplementary file 1 [file Data_Sheet_1.zip › 陈静数据分析/Model fit1.docx]

Model fit

|  | *χ2/df* | NFI | RFI | IFI | TLI | CFI | RMSEA |
| --- | --- | --- | --- | --- | --- | --- | --- |
| X→M→Y | 1.777 | 0.985 | 0.974 | 0.993 | 0.988 | 0.993 | 0.044 |

本研究中PTM的结构效度为*χ2/df* = 3.438，CMIN = 1017.588，RMSEA = 0.077，NFI = 0.835，RFI = 0.819，IFI = 0.877，TLI = 0.864，CF I= 0.876。说明在本研究中该量表的结构效度良好。

本研究中CD的结构效度为*χ2/df* = 2.737，CMIN = 744.397，RMSEA = 0.065，NFI = 0.846，RFI = 0.831，IFI = 0.897，TLI = 0.885，CF I= 0.896。说明在本研究中该量表的结构效度良好。

本研究中情绪调节量表的结构效度为*χ2/df* = 2.737，CMIN = 86.988，RMSEA = 0.062，NFI = 0.946，RFI = 0.928，IFI = 0.966，TLI = 0.955，CF I= 0.966。说明在本研究中该量表的结构效度优异。
